# Pharmacotherapy for Keloids and Hypertrophic Scars

**DOI:** 10.3390/ijms25094674

**Published:** 2024-04-25

**Authors:** Teruo Murakami, Sadayuki Shigeki

**Affiliations:** 1 Laboratory of Biopharmaceutics and Pharmacokinetics, Faculty of Pharmaceutical Sciences, Hiroshima International University, Higashi-Hiroshima 731-2631, Japan; genusmr@gmail.com; 2 Department of Rehabilitation, Faculty of Rehabilitation, Hiroshima International University, Higashi-Hiroshima 731-2631, Japan

**Keywords:** keloid, hypertrophic scars, pharmacotherapy, physical therapy, postoperative recurrence, multimodal combination therapy

## Abstract

Keloids (KD) and hypertrophic scars (HTS), which are quite raised and pigmented and have increased vascularization and cellularity, are formed due to the impaired healing process of cutaneous injuries in some individuals having family history and genetic factors. These scars decrease the quality of life (QOL) of patients greatly, due to the pain, itching, contracture, cosmetic problems, and so on, depending on the location of the scars. Treatment/prevention that will satisfy patients’ QOL is still under development. In this article, we review pharmacotherapy for treating KD and HTS, including the prevention of postsurgical recurrence (especially KD). Pharmacotherapy involves monotherapy using a single drug and combination pharmacotherapy using multiple drugs, where drugs are administered orally, topically and/or through intralesional injection. In addition, pharmacotherapy for KD/HTS is sometimes combined with surgical excision and/or with physical therapy such as cryotherapy, laser therapy, radiotherapy including brachytherapy, and silicone gel/sheeting. The results regarding the clinical effectiveness of each mono-pharmacotherapy for KD/HTS are not always consistent but rather scattered among researchers. Multimodal combination pharmacotherapy that targets multiple sites simultaneously is more effective than mono-pharmacotherapy. The literature was searched using PubMed, Google Scholar, and Online search engines.

## 1. Introduction

Three distinct but overlapping phases are involved in wound healing: the inflammatory, proliferative, and remodeling phases. However, the modulated healing process of reticular dermis injury, or excessive tissue response to dermis injury, induces keloids (KDs) or hypertrophic scars (HTSs). Both KD and HTS are raised and pigmented with increased vascularization and cellularity, and these scars accompany symptoms such as pain, itching, and contracture in patients. In addition, depending on the scars’ locations, the scars may greatly decrease the patient’s quality of life (QOL) due to being visually conspicuous and uncomfortable. In normal wound healing, collagen, one of the pro-inflammatory factors, is produced to repair the damaged tissue. In KDs and HTSs, the activity of transforming growth factor-β (TGF-β) is high, and there is an overproduction of collagen, with a number of fibroblasts and newly formed blood vessels, leading to raised, pigmented and thickened scar tissue. However, the biological properties are markedly different between KDs and HTSs. KDs grow and protrude beyond the borders of the original wound, but HTSs protrude within the borders of the original wound. The raised HTS eventually shrinks with time, and spontaneous healing would be expected someday. In contrast, KDs amplify with the lapse of time after wounding and spontaneous healing is not expected. Genetic factors are involved in KDs but not in HTSs [1,2,3,4,5]. To date, the exact mechanisms underlying KD and HTS formation are unclear, clinical options remain limited and these scars are still intractable diseases. There is no gold standard of treatment that provides complete satisfaction of the patient’s QOL so far; however, available treatments that show favorable results are increasing. For example, combination pharmacotherapy that uses multiple drugs, and/or pharmacotherapy combined with physical therapy such as cryotherapy, laser therapy, radiation therapy, and silicone/silicone gel sheeting have been reported to give better results compared to mono-pharmacotherapy [6,7,8,9].

In this article, we reviewed the difference in biological properties between KD and HTS, the pharmacological roles of various drugs used for treatments of KD and HTS (mono-pharmacotherapy), a combination of pharmacotherapy using multi-drugs, a combination of pharmacotherapy combined with physical therapy including surgical excision, and pharmacotherapy for the prevention of the postoperative recurrence of KDs and HTSs. Drugs for KDs and HTSs were searched using PubMed, Google Scholar and Online search engines, and a combination of keywords such as KD, HTS, treatment, management and/or prevention was applied. New drugs, such as antisense drugs and antibody drugs, that are still under investigation with few clinical reports at present, are also involved. In addition, drugs such as tranilast and saireito that are used only in specific regions are also involved. Tranilast (N-[3,4-dimethoxycinnamoyl]-anthranilic acid; Rizaben) is an orally administered anti-allergy drug approved for use in Japan and South Korea. Saireito, a Japanese herbal medicine administered orally, is also used to treat KDs and HTSs in Japan. Accordingly, clinical data on tranilast and saireito are published mostly in Japanese. Also, many herbal medicines are thought to be used only in specific regions to treat and/or prevent KDs/HTSs, and in such cases, the clinical data may be mostly published in the local languages, implying few English references. Finally, we discussed some points of pharmacotherapy to increase the clinical efficacy, because the reported clinical data of each pharmacotherapy are not always consistent among researchers, possibly due to the different doses of drugs, different formulations, different treatment periods, different evaluation methods of clinical efficacy, etc. Thus, it may be difficult to verify the accuracy of each piece of information; however, the information on various drugs, including promising drug candidates and commonly used drugs and their usage methods, would give valuable information relevant to developing safer and more clinically effective pharmacotherapy.

## 2. Comparison of Biological Characteristics between Keloids and Hypertrophic Scars

Cutaneous injury is induced by irritations such as burns, insect bites, skin piercing, surgery, tattoos, trauma, vaccination, and so on, and the wound is healed with time if the healing process is normal. In contrast, however, if the healing process were to be modulated, raised scars such as KDs and HTSs, with increased vascularization and cellularity, would be induced. The process of wound healing is divided into the following three stages: hemostasis/inflammatory, proliferation, and maturation/remodeling, where these healing stages overlap following a time sequence. During the healing process of the cutaneous injury, various proinflammatory factors, such as TGF-β1/β2, and the extracellular matrix (ECM), such as collagen, elastin, hyaluronic acid (HyA) and proteoglycans, are produced to repair wounds. If the healing process is modulated, various cytokines and ECM are overexpressed and KDs/HTSs with raised and firm scars are formed. As risk factors that promote these scars, various factors are involved, as follows: local factors (tension on the wound/scar), systemic factors (e.g., hypertension, pregnancy), genetic factors (sex, single-nucleotide polymorphisms, skin color, etc.), lifestyle factors involving consuming hot and spicy foods or taking hot baths, and so on [2,3,5,10,11,12,13]. Although both KDs and HTSs are produced mainly due to the overexpression of TGF-β1/β2 and collagen, clearly different biological properties are also involved in the two scar types. In Table 1, some properties such as the onset of scars, scar formation (appearance), scar sites, incidence, genetic factors, type of collagen, proinflammatory factors that enhance scars, the possibility of spontaneous regression, and the possibility of recurrence after surgical excision of scars are listed. Regarding the onset of scars, it is reported that KDs develop from 3 months to several years after injury, rarely mature, and do not follow the same pattern of evolution, stabilization and involution as normal and HTSs. In contrast, HTSs usually arise within 4–8 weeks after wound closure and develop over the next 6–8 months, after which progression usually halts and becomes quiescent. The raised scars of KD extend beyond the boundaries of the original wounds, but an HTS is contained within the boundaries of the original injury [13,14,15]. KDs are preferentially induced at sites with high skin tension, such as the chest, shoulder, upper back, posterior neck, cheeks, knees, and earlobes, but HTSs can be induced anywhere, and there are no predominant anatomical sites [16,17]. The occurrence of KDs and HTSs is mostly concentrated in individuals 9–20 years old (HTS: 77.2%; KD: 81.8%) in China; the male sex is a risk factor for HTSs (adjusted *p* < 0.001), and KDs are associated with family history (adjusted *p* < 0.050). Regarding the genetic factors, a strong genetic predisposition is reported for KD formation, and there is a high prevalence of KD development in certain ethnicities. Individuals from African, Hispanic, and Asian backgrounds have a higher likelihood of developing KDs when compared to Caucasians. KDs do not occur in patients with albinism, suggesting that melanocytes play a possible role in KD formation. In addition, the contribution of different alleles of human leukocyte antigen is also reported in KD formation [17,18,19,20]. Differences in the structures of the basement membrane zone, such as the collagen structure in the dermal layers, and the frequency of mast cells between HD, HTSs and normal skin are also reported [21]. The raised scars of KD and HTS are due to the overproduction of ECM, especially collagen, and the quantity and composition of collagen are different between KDs and HTSs. It has been reported that there is a 20-fold increase in collagen production in KDs and a 3-fold increase in HTSs, leading to a larger, abnormal-appearing scar [12]. When the synthesis of type I and type III collagens was examined in cultured skin fibroblasts from normal skin, normal scars, HTSs, and KDs, the ratio of type I/III collagen was significantly elevated in KDs compared to that in the other groups, in parallel with the increase in α1(I) procollagen mRNA compared to normal skin tissue [22]. In HTSs, collagen III scar/normal ratios are higher, but there is no difference in collagen I scar/normal ratios [3]. The reticular layer of KDs and HTSs contains inflammatory cells, increased numbers of fibroblasts, newly formed blood vessels, and collagen deposits. Also, in KDs, various proinflammatory factors such as interleukin (IL)-1α, IL-1β, IL-6, and tumour necrosis factor (TNF)-α are upregulated [2]. In HTS, the expression levels of IL-31, IL-31 receptor α (IL-31RA), and oncostatin M receptor (OSMR) are increased in post-burn HTS tissues compared with normal tissue [23,24]. Regarding the regression of scars, KDs having a higher density and proliferating activity of dermal fibroblasts continue to increase in volume and invade the surrounding tissue. In contrast, HTSs with a higher density and lower proliferating activity show a tendency towards spontaneous regression [25]. KDs are treated in many ways, including surgical excision monotherapy; however, the recurrence rate of KDs after surgical excision alone without postoperative preventive therapy is quite high (50–100%), although the recurrence rate of HTSs after surgical excision is low [11,26,27].

## 3. Pharmacotherapy for Keloids and Hypertrophic Scars

To treat KDs and HTSs, various treatment modalities have been employed involving pharmacotherapy, such as topical application using tape/plaster and ointment, intralesional injections, the oral administration of medicines, and treatments without pharmaceutics such as rest/fixation therapy (taping, silicone gel sheeting), compression therapy (bandages, supporters, garments), surgery, radiotherapy, laser therapy, make-up therapy, psychosocial health care, and so on [27]. In this article, we focused on pharmacotherapy for KDs and HTSs. The wound healing process is divided into the following three stages: the hemostasis/inflammatory stage, proliferation, and maturation/remodeling. In normal wound healing, a balance is achieved between the new tissue biosynthesis and degradation mediated by the apoptosis and remodeling of ECM. In contrast, in the excessive healing process of KDs and HTSs, a dysfunction of the regulatory mechanisms, that is, persistent inflammation, excessive collagen synthesis and/or deficient matrix degradation and remodeling, are induced [10]. The overproduction/activation of cytokines that enhance fibrosis, such as TGF-β1/β2, platelet-derived growth factor (PDGF), insulin-like growth factor (IGF-I), IL-4, IL-10, etc., as well as ECMs that enhance fibrosis such as collagen, fibronectin and glycosaminoglycans during the hemostasis/inflammatory and cell proliferation phases, in addition to the decreased production of TGF-β3, INF-γ, IL-12, collagenases, metalloproteinase (MMP), and so on, which attenuate fibrosis during the maturation/remodeling phase, are induced [3,5,10]. Drugs that are mainly used for the treatments of KDs and/or HSs have suppressing effects on the overproduction of fibrosis-enhancing cytokines such as TGF-β1/β2, and ECMs such as collagen. The drugs listed in Table 2 are angiotensin-converting enzyme (ACE) inhibitors, antiallergic agents, antisense drugs, antiviral cytokines, biocomponents, calcium-channel blockers, chemotherapeutics, hydrolytic enzymes, fat-soluble vitamins, immunomodulators, monoclonal antibodies, neurotoxins, peripheral vasodilators, photosensitizers, plant-based medicines, statins, and steroids.

### 3.1. ACE Inhibitors (Captopril, Enalapril, Losartan)

ACE inhibitors are generally used to treat high blood pressure and heart problems because these drugs can relax blood vessels by inhibiting ACE in the body and decreasing blood pressure. In KD/HTS treatments, ACE inhibitors reduce fibroblast proliferation, suppress collagen and TGF-β1 expression, and downregulate the phosphorylation of SMAD2/3 and TAK1, both in vitro and in vivo, resulting in the inhibition of scar formation by suppressing both TGF-β1/SMAD2/3 and TGF-β1/TAK1 pathways, where SMADs/Smads are signal transducers for receptors of the TGF-β superfamily [28,29,30]. In KD fibroblast cells, captopril at effective concentrations decreased all collagen metabolisms, the expression of TGF-β1, PDGF-BB and heat shock protein 47 (HSP47), and cellular proliferation [31]. In a patient with a postburn scar, the topical application of 5% captopril (twice/day for 6 weeks) showed a moderate to marked improvement in the KD lesion, and significantly decreased the redness, scaling, and itchiness [32]. In patients with HTSs and itching after treatment of second- or third-degree burns, the topical application of 1% enalapril ointment led to significantly smaller scars than scars in the placebo side, and significantly lower itching scores compared to the placebo group [33]. The efficacy of oral enalapril was also reported using a rabbit ear wounding model, in which the early oral application of enalapril following dermal injury reduced the formation of an HTS, probably because of its downregulatory effects on type III collagen production [34]. Treatment with losartan cream inhibited the expression of TGF-β1, collagen, and Smads, and decreased the phosphorylation of Smad in vitro and in vivo (mouse scar model) [29,35]. The efficacy of 5% losartan potassium ointment was examined in patients with HTSs and KDs, in which vascularity and pliability were significantly reduced by losartan treatment, and the Vancouver Scar Scale (VSS) scores (evaluation of scars from vascularity, height, pliability, and pigmentation) dropped significantly in both KD and HTS patients [36]. Regarding oral losartan treatment (started at post-burn day 1 and continued for 28 days), it was reported that the extensive graft loss seen in losartan-treated wounds is most likely responsible for the poor clinical outcome of full-thickness burn wounds, and losartan treatment should not be started before transplantation to prevent graft loss [37].

### 3.2. Antiallergic Agent (Tranilast)

Tranilast (Rizaben^®^, Kissei Pharmaceutical Co., Ltd., Tokyo, Japan) is an anti-allergic agent used to treat various inflammatory diseases such as allergic rhinitis, asthma, atopic dermatitis, bronchial asthma, allergic conjunctivitis, KDs and HTSs. Tranilast is approved in Japan and South Korea for the treatment of KDs and HTSs. As dosage formulations of tranilast, oral formulations, nasal sprays and eye drops are available. Tranilast suppresses type I allergic reactions by inhibiting the release of chemical mediators such as histamine and leukotrienes from mast cells and various inflammatory cells. It also inhibits the production of collagen, TGF-β, interferon (INF)-γ, IL-6, IL-10, IL-17, vascular endothelial growth factor (VEGF), MMP-2 and MMP-9, TNF-α, and some other angiogenic and inflammatory factors [38,39,40,41,42,43,44,45]. When the clinical effect and usefulness of tranilast (300 mg/day for 12 weeks) were evaluated in patients with KDs or HTSs, the final overall ratings of improvement, safety and usefulness were 66.7% (moderate or better), 82.9%, and 61.3% (moderate or better), respectively. In detail, more than 2 grades of improvement in itching and spontaneous pain were obtained in more than 40% of the patients, and the improvement in pressure pain was about 30%. In addition, more than 2 grades of improvement in the enlargement tendency and erythema of scars was obtained in about 50% and 30%, respectively. Adverse reactions such as mild digestive symptoms, which recovered after withdrawal or by taking additional medicaments, were reported in 11.4% [46]. Similarly, the oral administration of tranilast (5 mg/kg/day for 12 weeks) manifested significant improvements in 263 Japanese patients with KDs or HTSs [44,47]. In addition to oral administration, tranilast can be administered rectally [48], transdermally [49,50,51,52], and by eye drops, including on the corneal and eyelid skin [53,54,55,56]. When tranilast (12 mg in 1.5 mL of ethanol/water mixture) was delivered transdermally via an iontophoretic device (2 mA from the power supply module of an iontophoretic device for 30 min once a week) to the affected HTS parts, the patients’ complaints of pain and itching were reduced clearly after only one or two treatments, with some variations among patients. When the scars of HTS were excised within one or two hours after 30 min iontophoresis, tranilast was recovered in the scars in a range from 0.02% to 0.2% of the dose given, suggesting that iontophoretic delivery is more beneficial than the oral delivery and topical application of tranilast [49].

### 3.3. Antisense Drug (Antisense Oligodeoxynucleotides of TGF-β1, SMAD3, and TERT)

Antisense oligodeoxynucleotides, which seal the targeting gene, are designed to modulate the function (signaling) and/or expression of targeted sense RNA. TGF-β1 plays a central role in KD formation. Treatment with TGF-β1. antisense induced KD fibroblast apoptosis and inhibited KD fibroblast proliferation in vitro [57]. Also, TGF-β1 antisense treatment significantly increased the expression of hepatocyte growth factor/scatter factor (HGF/SF) in KD fibroblast cell culture [58], significantly downregulated the expression of SMAD2 and SMAD4 and the secretion of matrix MMPs, and upregulated the expressions of SMURF2, MMP-2, -3, -9, and -13, and MMP-1 and -2, but showed no effect on SMAD3 and SMAD6 in KD fibroblasts in vitro [59,60,61].

Topically applied TGF-β1. antisense preparations downregulated TGF-β1. protein expression levels and improved scar histology as determined by the scar elevation index in vivo [62]. The treatment of KD fibroblasts with cationic lipid nanoparticle-based SMAD3 antisense inhibited SMAD3, a primary inducer of fibrosis, and suppressed collagen type I production in KD fibroblasts, suggesting this delivery treatment has a therapeutic potential to suppress collagen deposition in fibrotic diseases [63]. Also, when KD fibroblasts were treated with human telomerase reverse transcriptase (TERT) antisense oligodeoxynucleotide (1.0 mol/L for 72 h), the fibroblasts’ growth was suppressed and the ability of proliferation decreased, during which time fibroblast apoptosis was induced, and the expressions of hTERT and bcl-2 mRNA were lower than those of the controlled group. The inhibition of telomerase activity in KD fibroblasts is an important pathway that may play a key role in anti-KD therapy [64].

### 3.4. Anti-Viral Cytokines (Interferons)

Interferons (IFNs) are a group of naturally occurring biological response modifiers that exhibit antiviral, antiproliferative, differentiating, and immune-enhancing properties [65]. Human peripheral blood mononuclear cells (PBMCs) activated with concanavalin A or lipopolysaccharide produce, respectively, lymphokines containing mainly IFN-γ or monokines containing primarily IFN-β, and these IFNs can inhibit collagen production [66]. Confluent cultures of keloidal fibroblasts produce more collagen (171%, 187%, and 204%), more glycosaminoglycans (153% and 141%), and less collagenase (26% and 31%) than normal fibroblasts. When keloidal fibroblasts were treated with IFN-α2b in vitro, the amounts of collagen, glycosaminoglycans, and normalized collagenase activity were 64–107%, 96–97%, and 86–96% of the control levels, respectively [67]. IFN-α, β, and γ suppress collagen synthesis by dermal fibroblasts. IFN-γ also suppresses collagen synthesis by myofibroblasts, synovial fibroblast-like cells, and type II collagen synthesis in human articular chondrocytes [68]. It was reported that IFN-α2b reduced collagen protein synthesis and type I messenger RNA levels in both HTS and normal fibroblasts after treatment, but these changes were apparent only after approximately 72 h, and HTS fibroblasts recovered completely from the effects of IFN-α2b on procollagen type I messenger RNA within 48 h of cessation of treatment. This suggests that the hypertrophic fibroblast may remain less sensitive to its effects [69]. In IFN-α2b-treated HTS fibroblasts, tissue inhibitors of MMP-1 and collagenase mRNA increased by 81% and 54%, respectively. In IFN-γ-treated fibroblasts, the tissue inhibitor of MMP-1 mRNA increased by 78% in HTS and 56% in normal dermal fibroblasts, but decreased collagenase mRNA by 59% and 42%, respectively [70]. Liposome-encapsulated IFN-α2b (2000 units per mL) significantly reduced the proliferation of dermal fibroblasts by 40%, and the levels of mRNA for type I by approximately 60% and for type III by approximately 30% procollagen [71]. The variation in the clinical efficacy of INF among researchers may come from the variation in the incorporation (distribution) of INF into the scar tissues. The greater incorporation (distribution) of INF is expected to exhibit higher pharmacological activities.

In clinical trials, intralesional injections of IFN-γ decreased the scars by at least 50% in dimension, as well as flattening them out and showing no serious toxic effects up to a dosage of 0.05 mg/week for 10 weeks [72]. Treatment with intralesional recombinant human IFN-γ (0.01 or 0.1 mg/one lesional site) demonstrated a reduction in size at the treated site, with an average reduction in height of 30% vs. 1.1% for control sites [68]. Similarly, the intralesional injection of IFNs showed impressive reductions in the size and collagen production of KD [73,74]. Patients with HTS were treated with systemic IFN-α2b, and they showed a general reduction in the total number of fibroblasts and myofibroblasts associated with a significant increase in the percentage of apoptotic cells compared with normal dermis from the same patient [75].

### 3.5. Calcium Antagonists (Verapamil)

In KDs and HTSs, the overproduction of ECM collagen and proteoglycans is observed, and the cellular secretion of macromolecules is a calcium-dependent process. Calcium antagonists such as VER and nifedipine can suppress ECM protein synthesis. Calcium antagonists inhibit transmembrane calcium influx, the growth and proliferation of vascular smooth muscle cells and fibroblasts, and the synthesis of ECM proteins (collagen, fibronectin, proteoglycans) [76,77,78]. VER enhances collagenase secretion in human skin fibroblasts, suggesting that VER enhances collagen I breakdown [79]. In addition, VER has an antioxidant activity, which enhances the production of nitric oxide. Nitric oxide promotes the proliferation of fibroblasts, keratinocytes, endothelial cells, and epithelial cells during wound healing [80]. The use of calcium antagonists in dermatology is mainly based on their properties as vasodilators and the inhibition of muscle contractions such as facial wrinkles and painful leiomyoma [81]. In addition, calcium antagonists can alter cell shape and induce procollagenase synthesis in KD and normal human dermal fibroblasts [82]. In in vitro studies, calcium antagonists have been found to reduce ECM production, induce procollagenase synthesis, and inhibit IL-6, VEGF, and the proliferation of fibroblasts [83].

In clinic, patients treated with topical VER presented good-quality scarring (80% of mammoplasty scars and 75.2% abdominoplasty scars), while no use of healing modulators showed 48 and 51.2% satisfaction, respectively, indicating that the use of topical VER can avoid the development of KDs and HTSs after plastic surgery [84]. VER was administered by intralesional injection (0.03 mg/kg, every 7 to 10 days, 6 sessions) in conjunction with pressure garments to improve the condition of the KDs and HTSs caused by the burn. VER was found to be very useful when the treatment efficacy was evaluated by various parameters such as pigmentation, thickness, vascularity, and flexibility. Also, no adverse effects were found [85]. In KD patients, VER was injected intralesionally at 3-week intervals until reaching the complete flattening of the lesion or for a maximum of 6 sessions, in which 55% of the patients showed excellent or good improvement with no significant side effects, except for pain at the site of injection. A significant decrease was observed in VEGF and MMP-9 expressions after VER treatment [86]. Earlobe KD is usually recalcitrant to treatment and has a high rate of recurrence. Recurrent earlobe KD was treated with a core fillet flap and intralesional VER injection. The results indicate that this treatment is a reliable and cost-effective method in the treatment of recurrent earlobe KDs, with a low rate of recurrence (28.6%) and high patient satisfaction (80%) [87]. The efficacy of the intralesional injection of VER (2.5 mg) was compared with that of TAC (40 mg) in the treatment of KD in an African population. TAC completely resolved pain and pruritus in 6 and 12 weeks, respectively, while there was no complete resolution among VER-treated patients. However, VER injection was comparably effective to TAC for small-size KDs [88]. Similarly, when the efficacy and safety of VER were compared with that of TAC, VER yielded fewer adverse events than TAC in the treatment of KDs and HTSs. These results indicate that intralesional VER can be used as a safer alternative to intralesional TAC [89,90,91,92]. Another group reported that the rates of reduction in the vascularity, pliability, height and width of the scar with TAC were faster than with VER, although adverse drug reactions were more prevalent with TAC than with VER [93]. It was also reported that no therapeutic event or significant improvement was seen in the VER treatment (2.5 mg/mL at three-week intervals for a total of 18 weeks), different from the cases of TAC treatment (40 mg/mL, the same dosing schedule to VER), in which VSS evaluated at the end of the 3-month follow up [94].

### 3.6. Chemotherapeutics (Bleomycin, 5-Fluorouracil, Mitomycin C, Paclitaxel, Tamoxifen)

Chemotherapeutic agents including alkylating agents, antimetabolites, topoisomerase inhibitors, antibiotics, mitotic inhibitors, and protein kinase inhibitors can induce apoptosis, autophagy and cell cycle arrest in tumor cells [95,96]. Chemotherapy-induced apoptosis establishes that cancer chemotherapeutic agents can induce programmed cell death in tissues [97]. Chemotherapeutics such as BLM, 5-FU, and mitomycin C (MMC), in addition to steroid injection, have been proposed as effective modalities for scar treatment and scar prevention after surgery, because these drugs target the fibroblasts in scar tissue, and induce apoptosis or modulate protein production [98,99].

#### 3.6.1. Bleomycin (BLM)

In clinic, to treat KDs and HTSs, BLM, an anticancer agent, was administered through multiple superficial puncture techniques. Complete flattening was observed in 44%, significant flattening in 22%, and adequate flattening in 14% of patients, but in 20%, flattening was not observed, and recurrence was seen in 14%. It was concluded that this treatment is the first-line treatment modality for the management of KDs and HTSs [100]. The usefulness of intralesional BLM to treat KDs and HTSs was also reported by many research groups. In these studies, BLM was administered via various techniques to decrease injection pain, including using a multiple-puncture method on the surface of the skin, electroporation, or needle-free jet injectors [101,102,103]. Separately, patients with KD were treated with intralesional BLM (average four times). Complete flattening reached 70.8%, and highly significant flattening reached 8.3%, in which the local side-effects were pains (100%), blisters (78.3%), ulceration (5.8%), and hyperpigmentation (56.7%). The recurrence rates of KDs 6, 12, 15 and 18 months after the last treatment were 3.8, 15.4, 45.5 and 50%, respectively [104]. Patients with KDs (*n* = 314) were treated with monthly BLM injections after surgical shave excision and then reepithelialization. In total, 87% of the patients were very satisfied with complete flattening, 11% were moderately satisfied with significant flattening, and 2% showed recurrences [105]. Patients with KDs and HTSs were treated with the intralesional injection of triamcinolone (20 mg/mL) or BLM (1.5 mg/mL) every 3 weeks for a maximum of six sessions. There was no significant difference in the efficacy between the two treatment groups, although the side effects induced were different. It was concluded that intralesional BLM is as effective as triamcinolone; however, BLM should be used carefully, due to adverse events such as pain, ulceration, and hyperpigmentation [106].

#### 3.6.2. Hydroxycamptothecin (HCPT)

Hydroxycamptothecin (HCPT), a natural plant alkaloid, has been proven to induce apoptosis in fibroblasts, in which endoplasmic reticulum stress response and mitochondrial dysfunction are involved [107]. In a postlaminectomy rabbit model, the implantation of HCPT liposomes in the laminectomy area inhibits collagen secretion and induces fibroblast apoptosis, which would prevent the adhesions of epidural scars [108]. Similarly, HCPT induced the apoptosis of fibroblasts and prevented intraarticular scar adhesion by activating the inositol-requiring kinase1 signal pathway in rabbits [109]. Abnormal microRNA (miR) 23b 3p expression has been detected in various types of fibrotic tissues that are present in different diseases. HCPT treatment notably increased miR 23b 3p expression levels and accelerated fibroblast apoptosis, indicating that the upregulation of miR 23b 3p expression induced by HCPT promotes fibroblast apoptosis [110]. 10,11-Methylenedioxycamptothecin (MD-CPT) inhibits KD. The transdermal delivery of MD-CPT loaded HyA nanoemulsions inhibited the proliferation of KD fibroblasts, without causing serious toxicity to normal skin fibroblasts [111].

#### 3.6.3. 5-Fluorouracil (5-FU)

Bulstrode KDs are fibrous lesions formed at the site of trauma due to types I and III collagen irregular production. 5-FU, a fluorinated pyrimidine analogue acting as an anti-metabolic agent, inhibits thymidylate synthase and interferes with ribonucleic acid (RNA) synthesis. In clinic, the intralesional injection of 5-FU exhibits satisfactory results in the treatment of KDs, causing a reduction in scar volume and symptom improvement (90% of the patients improved) [112]. 5-FU is a potent inhibitor of TGF-β/SMAD signalling, capable of blocking the TGF-β-induced, SMAD-driven upregulation of COL1A2 gene expression in a JNK-dependent manner [113]. In primary cell lines of KD fibroblasts, treatment with a low-dose 5-FU (as low as 1 mg/mL) induces the significant inhibition of proliferation, G2/M cell cycle arrest and apoptosis, but not immediate cell death in KD fibroblasts. These results support the use of low-dose 5-FU as a potential modality for treating KD scars [114]. 5-FU caused a dose-dependent, selective, and specific decrease in collagen production as a result of Dupuytren’s fibroblasts compared with noncollagenous protein synthesis. The treatment of fibroblasts with 5-FU selectively inhibited collagen synthesis, whereas 5-FU treatment did not affect procollagen types I and III mRNA [115]. When 5-FU was injected intralesionally into KDs at a dose of 50–150 mg per week for a maximum of 16 injections, more than 75% flattening of the KD was observed in 33.3% of the patients, and about half of the patients showed more than 50% flattening of the treated KD. The side effects were pain (all patients), hyperpigmentation (all patients) and ulceration (4.2% of patients) [116]. 5-FU is thought to be a safe and practical alternative for the treatment of KD and HTS [117]. The intralesional injection of 5-FU manifested a more than 50% improvement in 85% of patients with KD, although one patient did not respond favorably. Small and previously untreated lesions improved the most. Pain (all patients), hyperpigmentation (all patients), and tissue sloughing (30% of patients) were the main adverse effects. Recurrence was observed in 47% (9 of 19) of patients who responded to treatment within 1 year [118]. The topical application of 5-FU-loaded carboxymethyl chitosan nanoparticles showed a significant inhibitory effect on the human KD fibroblast to 16% [119]. A single needle-free pneumatic jet injection (PJI) containing 5-FU and TAC can significantly improve the height and pliability of HTS. PJI is favored by patients and may serve as a complement to conventional needle injections, especially for patients with needle phobia [120]. A bilayer-dissolving microneedle containing TAC and 5-FU exhibits biphasic release profiles. It causes the downregulation of mRNA and protein expression of collagen I (Col I) and TGF-β1 in HTS therapy [121].

#### 3.6.4. Mitomycin C (MMC)

Metabolites of MMC bind to DNA-crosslinking, which interferes with the synthesis of DNA, RNA and proteins. MMC is thus able to reduce fibroblast proliferation [122]. Continuous exposure to MMC causes fibroblast cell death within 7 days at all tested concentrations (concentration range from 0.0004 mg/mL to 4 mg/mL) at different rates depending on the MMC concentration in the culture medium [123]. Topical MMC (0.5 mg/mL) for 5 min delays the healing of surgical wounds in rats up to the fourth week following treatment [124]. After the excision of KD scars, a pledget with 1 cc of MMC 0.4 mg/mL was applied for 5 min. All patients were satisfied with the results, although the complete disappearance of the KD was observed only in two [125]. KD scars were excised and MMC (0.4 mg/mL) was applied for 5 min to the resected bed before the closure of the wound. MMC made no difference in the prevention of postoperative KD recurrence when applied topically [126]. Ten patients had all or part of their KD shave removed, and MMC (1 mg/mL) was applied topically for 3 min after hemostasis. This treatment of KD was effective in most patients [127]. Intralesional MMC (1 mg/mL) was effective in treating KDs and HTSs. Similar results are reported by many researchers, in which it was concluded that the topical application of MMC following shaving excision was safe and effective for the treatment of KDs and HTSs [128,129,130]. Both topical and intralesional MMC were effective in managing auricular KD, where topical MMC showed better VSS scores and patient satisfaction than intralesional injection [131].

#### 3.6.5. Paclitaxel (PCT)

Paclitaxel (PCT) kills cancer cells through the induction of apoptosis. PCT binds microtubules and causes the kinetic suppression (stabilization) of microtubule dynamics [132]. Also, PCT treatment suppresses the production of TNF-α, IL-6 and TGF-β and inhibits the expression of α-SMA and collagen I in human KD fibroblast. PCT-cholesterol-loaded liposome showed a better ability to inhibit cell proliferation, migration and invasion, and effectively promoted apoptosis and arrested cell cycle in G2/M phase compared to PCT alone [133]. In the rabbit’s ear model of HTS, PCT reduced the formation of HTS. Local necrosis was found in the rabbit ear models treated with a PCT solution of >400 mg/L [134].

#### 3.6.6. Tamoxifen (TAM)

Tamoxifen (TAM), a non-steroidal anti-estrogen that is usually used in treating breast cancer, decreases the expression of TGF-β1, with the consequent inhibitions of both fibroblast proliferation and collagen production [135]. In KD fibroblasts, TAM decreases KD fibroblast collagen synthesis by decreasing TGF-β production. At 4 µM of TAM, total TGF-β activity was decreased by 49% compared to the control, and at 8 µM of TAM, the activity of TGF-β decreased by 85% [136]. KD fibroblasts show increased TGF-β1 production compared with fetal fibroblasts. TAM improves wound healing in KD by decreasing the expression of TGF-β1 [137]. In normal adult human skin fibroblasts, at concentrations of TAM from 10 to 20 µM, the degree of lattice contraction was dose-dependent, but at concentrations from 50 to 100 µM, contraction in fibroblast-populated collagen lattices was completely inhibited. The dose- and time-dependent inhibition of contraction by fibroblasts suggests that TAM could have potential applicability in treating abnormal dermal scarring [138]. In clinic, TAM delays cellular proliferation rates of normal human dermal fibroblasts in a dose-dependent manner up to a concentration of 12 µg/mL, and higher concentrations, approaching 50 µg/mL, appear to have a toxic effect on cell growth. The analysis of growth factor production revealed decreased levels of basic FGF and VEGF, but no change in the levels of TGF-β1 [139]. Patients with a history of HTSs underwent surgery with different skin incisions, received TAM tablets postoperatively according to a standard protocol, and the development of HTSs was evaluated. It was concluded that TAM seems to be an effective agent in preventing the recurrence of HTSs after surgery [140]. The intralesional injection of TAM promotes the reduction in inflammatory stimulus and collagen fiber, as well as a significant reduction in the number of fibroblasts that produce collagen [141]. The topical application of TAM 2% ointment (twice daily for 8 weeks) increased angiogenesis and decreased the fibrotic tissue thickness, without changing the scar surface area in burned skin areas. This indicates that TAM can accelerate the wound healing process by reducing contracture and preventing HTSs and KDs formation [142].

### 3.7. Enzyme (Collagenase, Hyaluronidase)

In KDs/HTSs with raised and firm scars, ECMs such as collagen, elastin, HyA and proteoglycans are overproduced [143]. Both collagenase and hyaluronidase can produce low-molecular-weight fragments by hydrolyzing high-molecular-weight collagen (peptides) and HyA (glycosaminoglycans), respectively [144].

#### 3.7.1. Collagenase

It is reported that the intralesional injection of collagenase can degrade collagen fiber and decrease KD volume, although the volume may return to the same (or greater) levels with time. The side effects of collagenase injection are pain, swelling, blistering, ulceration and ecchymosis at the site of injection [145]. In clinic, patients with earlobe KDs were treated with the intralesional injection of collagenase and compression earrings daily for 12 months after injection. All patients had a decrease in the size of their earlobe KD by an average of 50%. Side effects included injection site swelling, tenderness, and one ulceration that spontaneously resolved within 2 weeks. It was concluded that intralesional collagenase followed by compression appears to be a safe and modestly effective treatment for earlobe KD [146].

#### 3.7.2. Hyaluronidase

In aesthetic plastic surgery, hyaluronidase is commonly used for over-correction and asymmetry, in which the dose of hyaluronidase is rather diverse and heterogeneous [147]. It is reported that the distribution, amounts, molecular weights and characteristics of HyA in scar tissue are different from those of normal tissue [21,148]. Low-molecular-weight fragments of HyA produced by hyaluronidase are known to stimulate angiogenesis and activate mesenchymal stem cells [144]. In dermatology, the use of hyaluronidase is efficacious for enhancing drug delivery to local sites including KDs/HTSs, the treatment of disorders associated with mucin deposition, and its potential uses in surgery [149]. In clinic, hyaluronidase is highly effective in eliminating HyA volume overproduction, and the intracutaneous injection of hyaluronidase can eliminate patient discomfort and inaesthetic lumps within a few hours [150]. The treatment of postoperative scars with hyaluronidase ointment significantly improves the function as well as the cosmetic appearance of scar tissues. The efficiency of hyaluronidase ointment was examined in the treatment of postoperative HTSs in patients with postoperative HTSs on different parts of the face, in which hyaluronidase was delivered transdermally with the help of iontophoresis. Scar conditions were assessed by VSS score. Total VSS score decreased to 5.14 ± 0.9 after 1 cycle of treatment and to 0.85 ± 0.9 after 2 cycles of treatment from 10 ± 1.5 before treatment [151]. In the treatment of KDs, intralesional TAC, intralesional TAC with hyaluronidase, and intralesional radiofrequency with TAC were effective modalities. Among these treatments, the combination of intralesional TAC and hyaluronidase was better than the other two as far as safety is concerned, with the fewest side effects [152].

### 3.8. Fat-Soluble Vitamins (Vitamin A, Vitamin D3, Vitamin E)

Vitamins, a natural constituent of human skin, are part of a system of antioxidants and protect the skin from oxidative stress. Products containing retinol (vitamin A), L-ascorbic acid (vitamin C), α-tocopherol (vitamin E), and niacinamide have potent antioxidant and anti-inflammatory properties. They are effective in the treatment of inflammatory dermatoses, acne, pigmentation disorders and wound healing [153]. In this section, the efficacies of lipophilic, fat-soluble vitamins such as vitamins A, D3 (cholecalciferol) and E in treating KD/HTS are reviewed.

#### 3.8.1. Vitamin A

Vitamin A deficiency retards repair. Retinoids (vitamers chemically related to vitamin A) restore steroid-retarded repair toward normal. Vitamin A tends to suppress fibroblasts in cell culture and stimulates steroid-treated macrophages to initiate reparative behaviors in tissue [154]. The stimulatory effect of vitamin A and retinoic acid on collagen accumulation and fibroplasia in healing wounds is due to fibroblast differentiation and enhanced collagen synthesis [155]. A review article reported that vitamin A functions mostly through nuclear retinoic acid receptors, retinoid X receptors, and peroxisome proliferator-activated receptors. Retinoids regulate the growth and differentiation of many cell types within the skin, and their deficiency leads to abnormal epithelial keratinization. In wounded tissue, vitamin A stimulates epidermal turnover, increases the rate of re-epithelialization, and restores epithelial structure [156].

In clinic, daily topical applications of a 0.05% retinoic acid solution reduce the size of KD/HTS scars in a range from slightly to markedly, and decrease complaints such as itching in most patients [157]. In human fibroblasts, retinoids, especially all-trans-retinoic acid (tretinoin), produce a marked reduction in fibroblast proliferation and collagen synthesis [158]. After burn injuries, scarred skin lacks elasticity, especially in HTSs. Topical treatment with tretinoin (all-trans retinoic acid) can improve the appearance and quality of the skin (i.e., texture, distensibility, colour and hydration), which would improve QOL for patients [159]. The use of prednisone (40 mg or greater) before or within three days of wounding inhibits wound healing, but vitamin A given topically or systemically reverses the inhibition of prednisone [160]. High-dose vitamin A, such as 10,000 IU/kg/day, but not low-dose such as 1000 IU/kg/day, significantly reverses the inhibitory effects of corticosteroids, whether given preoperatively or only postoperatively, on the healing of intestinal anastomoses [161]. The systemic administration of vitamin A improved wound healing in patients on chronic steroids. In the wound-healing model using steroid-treated rats, vitamin A applied for 10 min before wound closure and a gel foam sponge alone placed before wound closure both resulted in an increased breaking strength and tensile strength [162]. Glucocorticoids (corticosteroids) cause the dehiscence of surgical incisions, increased risks of wound infection, and the delayed healing of open wounds. Vitamin A restores the inflammatory response, and promotes epithelialization and the synthesis of collagen and ground substances, although vitamin A does not reverse the detrimental effects of glucocorticoids on wound contraction and infection [163]. In rats, the subcutaneous injection of methylprednisolone significantly decreases TGF-β and IGF-I levels in the wound fluid and hydroxyproline content in the tissue. Oral all-trans- and 9-cis-retinoic acid partially reverse the methylprednisolone-induced TGF-β and IGF-I decrease, and significantly increase hydroxyproline content toward normal levels. These data indicate that steroids and retinoids have antagonistic effects on growth factors and collagen deposition in wound healing [164].

#### 3.8.2. Vitamin D

Vitamin D3, a powerful anti-inflammatory agent, is made in the skin, and its production is influenced by various factors, of which the amount of melanin is a crucial one. An increase in pigmentation has been shown to decrease the amount of vitamin D3 synthesis in the skin. KD is more prevalent in populations with darkly pigmented skin, such as African Americans. In addition to the regulation of calcium homeostasis, vitamin D plays important roles in cell proliferation, differentiation, cancer progression, inflammation, and fibrosis. In KD fibroblasts, vitamin D slows the progression of tissue fibrosis and inhibits collagen synthesis in dermal fibrosis. In addition, vitamin D3 also reduces cellular proliferation, collagen synthesis and the induction of apoptosis in KD fibroblasts in a dose-dependent manner. The activities of vitamin D are dependent on the vitamin D receptor (VDR), that is, VDR reduces protein levels in most KD scars. The reduced VDR expression is returned to control values after vitamin D or calcium supplementation [165,166,167,168,169,170,171]. In KD fibroblasts, 1,25-dihydroxyvitamin D3 (1,25D) suppresses the expression of TGF-β1-induced collagen type I, fibronectin and α-SMA, and modulates plasminogen activator inhibitor-1 and matrix MMP-9 expression induced by TGF-β1 [172]. In clinic, 1,25(OH)_2_D3, an active form of vitamin D, inhibits the proliferation of KD fibroblasts, and there are correlations between vitamin D receptor polymorphism and KD formation. Thus, vitamin D may exert an antifibrotic effect partially mediated by matrix MMPs [173]. A highly significant reduction in VSS score was observed after treatment with intralesional vitamin D injection (200,000 IU/1 cm lesion, weekly, 3–4 sessions) in patients with KD [170]. It was reported that intralesional vitamin D injection is a promising treatment option for KD due to its easy availability, low cost and favorable safety profile [174].

#### 3.8.3. Vitamin E (α-Tocopherol)

Using silicon (polydimethylsiloxane) plates on the KD surface is effective in treating KD, because of the hyperhydration of subcutaneous tissue. In patients with HTSs or KDs, the treatment with vitamin E added to the silicon plate yielded a significantly higher score than the treatment with silicon plate alone at the end of both periods (1 and 2 months) [175]. Vitamin E (α-tocopherol) influences wound healing through cellular signalling and gene expression, and affects methicillin-resistant staphylococcus aureus (MRSA) infection, while vitamin E supplementation is beneficial for wound repair and immune functions [176].

### 3.9. Immunomodulator (Tacrolimus, *Imiquimod)*

Tacrolimus, a calcineurin inhibitor, is an immunosuppressant mainly used in preventing rejection after organ transplantation, and is also used for various inflammatory skin disorders. In skin disorders, tacrolimus is used via topical application for the short term, because the use of oral tacrolimus is limited due to its severe adverse effects such as infections, hypertension, and neurotoxicity [177]. Imiquimod is a new class of immune response stimulator, enhancing both the innate and acquired immune pathways (particularly T helper cell type 1-mediated immune responses) resulting in antiviral, antitumor and immunoregulatory activities, and it was first approved for the topical treatment of external genital and perianal warts [178].

#### 3.9.1. Tacrolimus

In KD fibroblasts, proliferation and migration are significantly higher than those in normal fibroblasts, and tacrolimus inhibits proliferation, migration and collagen production enhanced by TGF-β1. Tacrolimus also suppresses the increase in TGF-β receptor I and II expression in TGF-β1-treated KD fibroblasts, indicating that tacrolimus effectively blocks the TGF-β/Smad signaling pathway in KD fibroblasts by downregulating TGF-β receptors [179]. In addition, tacrolimus significantly downregulates the expression of the human angiogenetic factors VEGF-A, FGF-2, PDGF-β, and TGF-β1 in human umbilical vascular endothelial cells and HTS fibroblasts. The tacrolimus-mediated inhibition of angiogenesis can decrease the gene expression of crucial fibrotic markers, including α-SMA and collagens 1 and 3 [180].

In a rabbit ear model, the intradermal injection of tacrolimus (0.5 mg/cm^2^) was found to be effective in preventing KDs and HTSs, without inducing general or local side effects [181]. Also, topical tacrolimus 0.03% and 0.1% ointments reduced the severity of inflammatory changes and positively altered the macroscopic aspect of the scar in the short term (30 days) in a rabbit ear model [182]. Similarly, tacrolimus 0.3% ointment was effective in suppressing TGF-β and SMA, reducing mucin, and improving the quality of collagen fibers and the density of elastic fibers in a rabbit ear HTS model [183]. Tacrolimus is commonly used in the treatment of psoriasis; however, its clinical application is limited by its transdermal drug delivery rate, etc. A HyA-based delivery system named blocking patch (BP) was developed to increase the transdermal delivery of tacrolimus. The BP loaded with tacrolimus showed a satisfactory transdermal release ability in vivo, and showed a good anti-inflammatory ability in mouse psoriasis-like dermatitis [184].

#### 3.9.2. Imiquimod

Imiquimod and its metabolite induce IFN-α in human blood cells and IL-1, IL-6, IL-8, and TNF-α in human PBMC cultures in vitro [185,186]. In addition, imiquimod also induces IL-1α, IL-1 receptor antagonist, IL-1β, IL-10, granulocyte-macrophage colony-stimulating factor (GM-CSF), granulocyte CSF (G-CSF), and macrophage inflammatory protein-1α in human blood cells [187]. The induction of various cytokines including INF-α, INF-stimulated genes, and IL-6 by imiquimod has also been reported using mice [188,189].

In clinic, imiquimod 5% cream was applied once daily at bedtime for 8 weeks. No recurrence was observed while on imiquimod application, but all KD completely recurred within 4 weeks of stopping imiquimod [190]. Topical imiquimod is frequently used to prevent the recurrence of KD after the surgical excision of KD, as described later.

### 3.10. Monoclonal Antibody (Dupilumab, Anti-TGF-β1 Antibody, Anti-VEGF-A Antibody)

Antibody therapy, also known as immunotherapy, using a monoclonal or polyclonal (multispecific) antibody is a type of medical treatment that targets specific proteins involved in disease processes and marks them for destruction by the immune system in the body. Antibody-based proteins are utilized for the analysis, purification, and enrichment of target proteins, and to mediate or modulate physiological responses [191]. In clinic, antibody therapy targeting antigens is one of the more important biological therapeutics, due in large part to the stability, specificity (targetability), and adaptability of the antibody framework. Derivatives of the monoclonal antibody format such as bispecific antibodies, antibody–drug conjugates, and antibody fragments have demonstrated efficacy in treating human disease, particularly in the fields of immunology and oncology [192]. KD is a fibroproliferative skin disorder that is caused by prolonged inflammation after cutaneous injury, and is characterized by collagen accumulation and blood vessel proliferation (enhanced angiogenesis) in the reticular layer of the dermis [193]. Cytokines such as TGF-β, IL-6, matrix MMP, IGF-1, and B cells are found in KD or HTS tissues. Developing biological antibodies targeting these cytokines could be a potential strategy for preventing and treating KDs/HTSs [194].

#### 3.10.1. Dupilumub

IL-4 and IL-13, signature type 2 cytokines, exert their actions by binding to two types of receptors sharing the IL-4R α chain (IL-4Rα). Since IL-4 and IL-13 play important roles in the pathogenesis of allergic diseases, blocking both the IL-4 and IL-13 signals is a powerful and effective strategy for treating allergic diseases. Dupilumab (Dupixent^®^) is a fully human monoclonal antibody that recognizes IL-4Rα and blocks both the IL-4 and IL-13 signals [195,196,197,198,199]. In clinic, a patient with numerous disfiguring HTSs or KDs after suffering from a severe herpes zoster infection was treated with dupilumub orally, and many scars flattened, but several scars further developed. When the large and most recalcitrant KD was further treated by an intralesional injection of dupilumub, an even more dramatic improvement was noted in 2 months [198]. Similarly, a patient with several KDs, who responded to oral tetracycline and then intralesional corticosteroids partially, was treated with subcutaneous dupilumab (every two weeks). The lesions appeared less elevated and erythematous with an increase in treatments [199]. In contrast, when two patients with diffuse KD were treated with dupilumab for three months, no improvement was observed, and one patient experienced clinical worsening. It was concluded that further studies are needed to investigate the utility of Th2 cytokine blockade as a potential treatment option for KDs [197].

#### 3.10.2. Anti-TGF-β1 Antibody

In cultured KD fibroblasts, the productions of type 1 collagen, matrix MMP-1, MMP-2, and tissue inhibitor of TIMP-1 were increased by 3-fold, 6-fold, 2.4-fold, and 2-fold compared to normal dermal fibroblasts, respectively. KD fibroblasts also showed a 2.5-fold increase in migratory activity. The addition of anti-TGF-β1 antibodies to the KD fibroblast cultures reduced the increased production and migration activity to the levels of normal dermal fibroblasts [200]. In an animal study, rabbit ear wounds were treated intradermally with anti-TGF-β1, 2, and 3 antibodies at early, middle, and late time points. Treated wounds from the early treatment group displayed delayed wound healing, with no reduction in scar hypertrophy. Both the middle and late treatment groups (beginning 7 days after wounding) showed a significant decrease in scar hypertrophy [201,202]. In a review article, it was reported that the use of IFN-α2b showed an 18% recurrence rate when applied to postsurgical excised KD. Imiquimod 5% lowered the recurrence rate of post-shaved KDs to 37.5% at 6 months and to 0% at a 12-month follow-up. TGF-β1 antisense oligonucleotides have shown effective and long-lasting inhibition of TGF-β-mediated scarring in vitro as well as in animal models. Daily injections of neutralizing antibodies against TGF-β1 and TGF-β2 showed successful reductions in scarring [203].

#### 3.10.3. Anti-VEGF-A Antibody

VEGF-A, an antiangiogenic IgG, is a key cytokine in the development of normal blood vessels, as well as the development of vessels in tumors and other tissues undergoing abnormal angiogenesis. VEGF also plays an important role in regulating scar tissue production, and a high VEGF level is linked with scar formation in normal, HTS, and KD scars, while the inhibition of VEGF resulted in scar tissue deposition [204]. In an in-vitro study, anti-TGF-β1, PDGF, anti-endothelin 1 (ET-1), anti-VEGF, and anti-basic FGF neutralizing antibodies were individually added to the culture medium of capillary endothelial cells. The endothelial dysfunction occurring in HTS contributes to fibroblast inhibition and scar regression, and reduced TGF-β1, PDGF, and basic FGF levels played more important roles in these processes than VEGF and endothelin 1 (ET-1) [205]. In an animal study, the efficacy of anti-VEGF antibody-modified liposome gels (PAE-BEV-lip gels) in the prevention and treatment of HTS was evaluated, in which PAE-BEV-lip gels exhibited a slower transdermal delivery rate, a remarkable dermal retention effect, and superior bioavailability in the rabbit ears. The treatment with topical PAE-BEV-lip gels showed a lower scar proliferation rate, fewer and looser collagenous fibers and fibromyocytes, more regular chondrocytes, less calcified tissue and fewer inflammatory cells compared to other groups. PAE-BEV-lip exhibited definite effects on the prevention and treatment of HTS in rabbit ears [206]. In clinic, anti-VEGF antibodies have demonstrated therapeutic utility in blocking VEGF-induced angiogenesis [207,208].

### 3.11. Neurotoxin (Botulinum Toxin, BTX)

BTX is a potent neurotoxin protein derived from the *Clostridium botulinum* bacterium, and it inhibits the release of acetylcholine at the neuromuscular junction. The injection of small quantities of BTX into specific overactive muscles causes localized muscle relaxation that smooths the overlying skin and reduces wrinkles [209]. BTX type-A (BTX-A) is also used for the treatment and prevention of KDs and HTSs in a monotherapy or combined therapy. The mechanism of BTX-A’s action is not yet clarified, but BTX-A involves action on wound tension, action on collagen, and action on fibroblasts [210]. In KD fibroblasts in vitro, the addition of BTX-A altered the expression levels of S100 calcium-binding protein A4, TGF-β1, VEGF, MMP-1, and platelet-derived growth factor subunit A (PDGFA) genes [211]. BTX-A inhibited the proliferation and differentiation into myofibroblasts [212]. It was also reported that BTX-A may reduce skin fibrosis by decreasing fibroblast proliferation, modulating the activity of TGF-β, and reducing the transcription and expression of profibrotic cytokines in KD-derived and HTS-derived dermal fibroblasts [213]. In the KD tissues, the expressions of myofibroblast markers, α-SMA, collagen I, and collagen III are increased, and BTX-A reduces the expression of α-SMA, collagen I and collagen III, enhances the expression of adipocyte markers, PPARγ and C/EBPα, and increases the accumulation of lipid droplets [214].

In clinic, patients with KD received either intralesional corticosteroid (group A) or intralesional BTX-A (group B). A significant decrease in the volume, height, and redness score of the lesions (A = B), and a significant softening of lesions (A > B), were observed, whereby all patients mentioned a significant reduction in their subjective complaints (A < B). Skin atrophy and telangiectasia were evident in three patients (25%) of group A, indicating that intralesional BTX-A exhibits higher efficacy and safety, and a comparable improvement of the objective parameters, as compared with intralesional corticosteroid [215]. BTX-A was reported to be a suitable potential therapy for the prevention of HTS. The scar width, patient satisfaction and visual analysis scores were all significantly different between the BTX-A group and the control (non-BTX-A used) group [216]. Similarly, BTX-A was more effective than non-treatment in preventing postoperative scars, including KD/HTS, and improving the cosmetic appearance of facial scars for East Asians [217]. The clinical efficacies of intralesional TAC, BTX-A and their combination for the treatment of KD lesions were compared. The combined injection of intralesional steroids with BTX-A appears to be superior to either therapy alone, and offers the best benefit of a safer and more efficacious response with fewer side effects [218]. When the clinical efficacies of intralesional BTX-A and 5-FU applied in KD treatment were compared, BTX-A achieved a better flattening of the lesions than 5-FU. In the BTX-A-treated group, there was no statistically significant difference between the clinical response in small lesions compared to medium and large ones, although, in the 5-FU treatment, small and medium lesions showed significantly better response than larger ones [219]. In the treatments of KDs and HTSs, the combination of corticosteroid with BTX-A was more effective than corticosteroid alone, as evaluated by VAS score, VSS score, scar thickness, itching degree and patient satisfaction [220]. In children with post-burn KDs/HTSs, the intralesional injection of BTX-A (every month for 6 months) significantly improved the associated itching, pain, pliability, erythema, and thickness of the scars as compared with control (no treatment) [221]. In a review article, BTX-A was injected into KD and HTS (1 session/month) for three sessions, and the clinical efficacy was evaluated by VSS, Observer Scar Assessment Scale (OSAS) and Patient Scar Assessment Scale (PSAS) scores. It was concluded that BTX-A could be a good therapeutic approach for the management of KDs and HTSs, with significant clinical and histologic improvement [222,223].

### 3.12. Peripheral Vasodilator (Pentoxifylline)

Pentoxifylline (PTF), an analogue of the methylxanthine theobromine, is a hemorheological agent with primary actions that include increasing erythrocyte flexibility, reducing blood viscosity and increasing microcirculatory flow and tissue perfusion, which improves the supply of oxygen to the ischemic muscles of the limbs [224,225]. PTF was initially developed for use in patients with intermittent claudication due to chronic occlusive arterial disease of the extremities. Thereafter, this drug has also been used to treat various dermatological diseases including peripheral vascular disease, vasculitis and vasculopathy, pigmented purpuric dermatosis, necrobiosis, KD, HTS, etc. [226]. PTF inhibits the proliferation of fibroblasts, collagen, glycosaminoglycan and fibronectin production, and increases collagenase activity in normal human dermal fibroblasts in vitro [67,227]. The inhibitory effects of PTF on proliferation and glycosaminoglycan synthesis were also observed in cultured fibroblasts obtained from patients with Graves’ ophthalmopathy and pretibial myxoedema [228]. In addition, PTF enacted a dose-dependent inhibition of contraction and reduced proliferation in fibroblasts isolated from mature burn scars [229]. In a culture of human fibroblasts derived from post-burn scars, PTF (1 mg/mL) decreased the cell number proliferation and contraction of fibroblasts, and selectively inhibited collagen III synthesis, although the inhibition of type I collagen synthesis was more evident in the non-scarred skin group [230]. Also, PTF inhibited the proliferation and rate of collagen synthesis of fibroblasts from KD, scleroderma, and morphea in vitro [231]. PTF/chitosan films (PTF/CSF) were developed for healing cutaneous wounds. In the wounds of mice, PTF/CSF with a higher concentration of PTF (4 mg/mL) reduced the area up to 60% on day 2 [232]. In clinic, burned patients received intralesional PTF as an adjuvant treatment for perioral post-burn HTSs. Intralesional PTF (1 mg/mL, five sessions with weekly intervals) were effective in treating post-burn HTS [230,233]. The efficacy of KD treatment was compared among intralesional PTF, TAC, and their combination. A combination of PTF and TAC produced significantly better results with a lower risk of TAC-induced side effects [234].

### 3.13. Photosensitizer Prodrug (5-Aminolevulinic Acid, Methyl Aminolevulinate)

Photodynamic therapy (PDT), a therapeutically promising method that involves the combined action of photosensitizers, oxygen, and light, has emerged as a therapeutically promising method for treating a broad variety of solid tumors and infectious diseases. In treating skin scars, PDT stimulates wound healing by enhancing re-epithelialization and promoting angiogenesis, as well as modulating skin homeostasis [235]. In a recent review article, it was reported that PDT using 5-aminolevulinic acid (5-ALA) as a photosensitizer has been developed as a promising noninvasive treatment for skin wounds such as skin cancer, chronic leg ulcers, and erosive pustular skin diseases, in which 20% 5-ALA solution, a wavelength between 600 nm and 670 nm (red light), an energy density of 120 J/cm^2^, a frequency of once per week and three sessions were the most widely selected therapeutic parameters [236].

5-ALA and methyl aminolevulinate (M-AL) are prodrugs and are metabolized intracellularly to form the photosensitizing molecule protoporphyrin (PpIX). When PpIX is activated by visible red light, cytotoxic ROS and free radicals are generated. This phototoxic effect can cause malignant and non-malignant hyperproliferative tissue to be destroyed, decrease in size, and eventually disappear [235,237]. In addition, topical ALA-induced PDT stimulates wound healing by enhancing re-epithelialization, promoting angiogenesis, as well as modulating skin homeostasis [238]. In an in vitro study, the cytotoxic effect of PDT at 5 J/cm^2^ and 10 J/cm^2^ of red light (633 ± 3 nm) with and without the degenerate wave on KD fibroblasts was evaluated using 5-ALA and M-AL. The cytotoxic effect of PDT on KD fibroblasts was found to be enhanced significantly by combining with degenerate wave stimulation [239]. The cytotoxic effect of PDT on KD fibroblasts obtained from different lesional sites (top, middle and margin) was evaluated by using M-AL and 5-ALA as precursors of intracellular photosensitizer. Cytotoxicity post-PDT in KD fibroblasts was dependent on the lesional site, the precursor of photosensitizer and te light energy, although PDT was effective for site-targeted KD therapy [240]. 5-ALA-loaded nanoethosome (5-ALA-ES) gels were prepared. 5-ALA-ES was found to increase the transdermal delivery in vitro and penetration into rabbit HTS tissue of 5-ALA in vivo. The efficacy of PDT was assessed using 5-ALA-ES gels and rabbit HTS models. The PDT of 5-ALA-ES gels improved HTSs by promoting the apoptosis of HTSs’ fibroblast, remodeling collagen fibers and increasing MMP3 expression [241]. The HTS fibroblasts treated with 5-ALA-PDT were examined. 5-ALA-PDT inhibited fibroblast contraction and promoted cell death by inhibiting the activation of the TGF-β1 signaling pathway that mediates HTS formation [242].

In clinic, a patient with KD, having a history of 4 years of negative response to various conventional treatments including topical silicone gel sheets, steroid cream, steroid injection and surgical excision, was treated with PDT using M-AL over 5 months. The size of the KD scar was significantly reduced, and the surface became flattened and smooth with an acceptable cosmetic outcome [243]. The efficacy of M-AL-PDT was evaluated in patients with KDs under three different conditions: existing KD scar, post-surgical debulking and post-total surgical excision. As a result, PDT reduced scar formation in KDs by decreasing blood flow, increasing pliability, and decreasing collagen and hemoglobin levels. Only 1 patient out of 20 patients experienced a recurrence of KD at a 9-month follow-up [244]. Severe HTS induced by facial hidradenitis suppurativa was treated with a high-concentration single dose of 5-ALA-PDT. The treatment could improve severe HTS effectively and safely, and no recurrence was observed up to 11 months of follow-up [245]. The complex of 5-ALA and HyA exhibited markedly enhanced skin retention and the increased generation and accumulation of endogenous photosensitizer (protoporphyrin IX) in vitro. In clinic, the 5-ALA-HyA complex effectively reduced the scar thickness and elevation index, and the scar was closely matched to the unwounded tissues. In addition, 5-ALA-HyA treatment markedly downregulated the gene expression levels of α-SMA and TGF-β1 [246]. The clinical efficacy, recurrence rate and safety of 5-ALA-PDT combined with microneedle or CO_2_ lattice laser were evaluated in comparison with intralesional betamethasone injection in the treatment of hypertrophic acne scar patients. Both PDTs showed equivalent clinical effects with intralesional betamethasone injection, but a lower recurrence rate within 6 months of the follow-up period [227]. A patient received 5-ALA-PDT on the first postoperative day, once a week for five sessions. At a two-year follow-up, the 5-ALA-PDT at an early stage was found to decrease vascular density and improve ECM deposition [247]. The clinical efficacy and safety of 5-ALA-PDT combined with 5-FU injection and TAC solution in the treatment of acne HTSs were evaluated in patients. After treatment, the scar lesions were reduced and flattened, the scars became soft, and there was no recurrence after 6 months of follow-up [248].

### 3.14. Plant-Based Medicine (Aloe Vera, Centella asiatica, Curcuminoids (Curcumin), Green Tea (Catechins), Hyperforin, Loureirin A/B, Onion Extract (Quercetin), Resveratrol, Saireito, Shikonin, Emodin, Glabridin, Kaempferol, Tripterine, Wubeizi)

Various plant-based medicines including pure and crude materials originating from plant sources are widely used to treat various diseases, including skin wounds such as KDs and HTSs, as well as cancers, in clinical settings. Many locally available plant-based medicines for KD and HTS treatments are reported from various regions, such as: Africa [249], the Balkan region (Southeast Europe) [231], the South Balkan and East Mediterranean region [250], China [251,252], the Georgia–Turkey border [253], Japan [254], Salta province (Argentina) [255], and southeastern Serbia [256]. Some examples of botanical sources of traditional Chinese medicine and their main active compounds effective for the treatment of HTSs are reported, as follows: *Alpinia officinarum* Hance (Galangin), *Centella asiatica* (L.) Urb., *Rheum palmatum* L. (emodin), *Panax ginseng* C.A. Mey. (Ginsenoside Rb1), *Scutellaria baicalensis* Georgi (baicalin), *Ginkgo biloba* L. (quercetin), *Conioselinum anthriscoides* “Chuanxiong” (essential oil), *Salvia miltiorrhiza* Bunge (cryptotanshinone), *Taxus wallichiana* Zucc. (PCT), *Stephania tetrandra* S. Moore (tetrandrine), and *Kaempferia galanga* L. (kaempferol) [252]. In this section, some active compounds and/or crude materials (extracts) that are available for the treatment of KDs/HTSs and originating from plant sources are reviewed.

#### 3.14.1. Aloe Vera

*Aloe vera* (L.) Burm. f. (Liliaceae family) is a well-known traditional medicinal plant that is used around the world, due to its potential antioxidant, anti-inflammatory, and wound-healing activities [257]. Aloe vera is composed of essential constituents beneficial for the wound healing process, such as amino acids, vitamins C and E, and zinc, and the main bioactive polysaccharide of aloe vera is reported to be acemannan [258,259]. In an in vitro study using normal human dermal fibroblast cells, a synergistic effect on cutaneous wound healing that targets microfibril-associated glycoprotein 4 was observed between aloe vera flower and aloe gel [257].

In clinic, one side of the abraded face was treated with standard polyethylene oxide gel wound dressings, and the other side with a polyethylene oxide gel dressing saturated with stabilized aloe vera. Wound healing was approximately 72 h faster at the aloe site, possibly due to the reduction in bacterial contamination, subsequent KD formation, and/or pigmentary changes [260]. The efficacy of silicone gel containing 15% herbal extracts for the prevention or amelioration of HTS was compared among allium cepa extract, centella asiatica extract, aloe vera extract and paper mulberry extract in patients who underwent median sternotomy. The silicone gel plus herbal extract gel significantly improved scar amelioration in terms of height and pliability when compared to the placebo [261]. Adding aloe vera to wound dressings such as silicone gel sheets, soft paraffin, and polyester dressings has become an ideal approach [262,263]. The efficacy of using silicone gel containing either aloe vera or onion extract to prevent postoperative KDs and HTSs was compared in patients who had undergone surgery. Both silicone gel sheets containing aloe vera or onion extract were effective in preventing postoperative scars. [262]. Some clinical data regarding the efficacy of aloe vera for treating burns are reviewed [263].

#### 3.14.2. *Centella asiatica* (Asiaticoside, Asiatic Acid, Madecasosside, Madecassic Acid)

*Centella asiatica* is a medicinal plant that was already used as a “panacea” 3000 years ago. This plant contains some pentacyclic triterpenes as active compounds, such as asiaticoside, madecasosside, asiatic acid and madecassic acid, in which asiatic acid and madecassic acid are the aglycones of asiaticoside and madecassoside, respectively, that are commonly used in wound healing. Among these compounds, asiaticoside and madecassoside are the marker compounds of centella asiatica (Indian pennywort) in the Chinese Pharmacopoeia, and these triterpene compounds have various pharmacological properties, including wound healing, anti-inflammatory, anti-oxidant, anti-allergic, and anti-fibrotic activities. Thus, these compounds are used extensively in treating skin abnormalities, including burn injuries, and are considered cosmetically beneficial for their role in anti-aging, skin hydration, collagen synthesis, UV protection and curing scars [264]. In KD fibroblasts, asiaticoside decreased fibroblast proliferation in a time- and dose-dependent manner, inhibited type I and type III collagen protein and mRNA expressions, reduced the expression of both TGF-βRI and TGF-βRII at the transcriptional and translational level, and increased the expression of Smad7 protein and mRNA. Asiatic acid inhibits TGF-β1-induced collagen expression, Smad 2/3 phosphorylation and plasminogen activator inhibitor-1 (PAI-1) expression in human KD fibroblasts through PPAR-γ activation, suggesting that asiatic acid is one of the active constituents responsible for KD management [265,266]. Madecassoside also suppresses the migration of fibroblasts from KD, enhances wound healing and diminishes KD formation, indicating this compound could be of great use in the treatment and/or prevention of HTSs and KDs [267]. These activities of asiaticoside and madecassoside indicate that Centella asiatica could be of potential use in the treatment and/or prevention of HTS and KD [265,268].

In clinic, the efficacy of using Centella asiatica extract in cream for the prevention of scar development at the split-thickness skin graft donor site was evaluated, in which cream was applied at least 2 weeks after epithelialization was completed. Centella cream improved pigmentation parameters and comparative total VSS scores [269]. The effect of supplementation with centellicum^®^ (Horphag Research Ltd., Geneva, Switzerland), a natural extract of Centella asiatica (Gotu Kola), on the healing of surgical wounds was evaluated in subjects with previous HTS or KD. It was concluded that supplementation with Centellicum^®^ is safe and does not interfere with other concomitant treatments. It is well tolerated and its compliance with treatment is optimal [270]. Recently, asiatic acid-entrapped transfersomes gel (AATG) was prepared for the treatment of HTS, and AATG indicated no adverse skin reaction, a significant reduction in melanin index and an increase in net skin elasticity at 2, 4, and 8 weeks [271].

#### 3.14.3. Curcuminoids (Curcumin)

Curcumin, a spice found in turmeric, is widely used due to its anti-inflammatory and antioxidant activities. In nature, curcuminoid is composed of curcumin, demethoxycurcumin and bisdemethoxycurcumin, and curcuminoids have been found to inhibit fibrosis. Curcumin can significantly inhibit cell proliferation and collagen type I synthesis in fibroblasts at higher concentrations (50, 100 µmol/L), although at a low concentration (12.5 µmol/L), curcumin showed a cell proliferation-enhancing trend [272]. In an in vitro study using KD fibroblasts, the excessive production of ECM in the KD fibroblasts and the level of TGF-β1/p-SMAD-2 was blocked by the cellular uptake of curcumin in a dose-dependent manner [273]. In an animal study, crude or pure curcumin (6 μg/kg, 30 μg/kg, or 60 μg/kg) dissolved in 1% ethanol was administered intravenously to rabbits before wounding. Treatment with pure curcumin significantly promoted nonischemic wound healing and increased re-epithelialization and granulation tissue formation in association with significant decreases in pro-inflammatory cytokines IL-1 and IL-6, as well as the chemokine IL-8 [274].

#### 3.14.4. Green Tea Extract (Catechins Especially (-)-Epigallocatechin-3-gallate, EGCG)

Green tea (*Camellia sinensis*), unfermented tea, and the constituent catechins have versatile pharmacological activities, such as antioxidant, anticancer, hypoglycemic, antibacterial, antiviral, neuroprotective, angiogenesis and antifibrotic properties. For example, green tea can assist in the treatment of diabetes, Alzheimer’s disease, infectious disease, oral cancer, and dermatitis, and exerts an important role in health care and disease prevention in daily life [275]. The important components in green tea that show antimicrobial properties are the catechins, and the four main catechins are (-)-epicatechin (EC), (-)-epicatechin-3-gallate (ECG), (-)-epigallocatechin (EGC), and (-)-epigallocatechin-3-gallate (EGCG) [275]. Among these catechins, EGCG has multiple potent effects on human pathological and physiological processes such as anticancer, antioxidant, anti-inflammatory, anticollagenase, and antifibrosis effects [276].

Green tea extract and EGCG alone inhibit mast cell-stimulated type I collagen expression, possibly by interfering with the PI-3K/Akt/mTOR signalling pathway in KD fibroblasts [277]. Also, EGCG suppresses growth and collagen production in the in vivo KD model, demonstrating that EGCG can suppress the pathological characteristics of KD through inhibition of the STAT3-signaling pathway [278]. In a rabbit ear HTS model, EGCG significantly inhibited HTS formation and the mRNA expression of TGF-β1, Col I, Col III, α-SMA, and eNOS. This suggests that EGCG is a useful therapeutic drug for HTS, acting by inhibiting fibrotic gene expression and suppressing angiogenesis [279].

In the in vivo KD model, EGCG treatment also suppressed growth and collagen production, demonstrating that EGCG suppresses the pathological characteristics of KD through the inhibition of the STAT3-signaling pathway [278]. In human KD tissue in situ, EGCG reduced KD volume significantly (40% by week 4), increased apoptosis (≥40% from weeks 1 to 4), and decreased proliferation (≤17% by week 2). In ex vivo evaluation, EGCG induced epidermal shrinkage, reduced collagen-I and -III at mRNA and protein levels, depleted 98% of KD-associated mast cells, and reduced the percentage of both cellularity and blood vessel count by week 4 [280].

#### 3.14.5. Hyperforin

*Hypericum* (*H.*) *perforatum* (*Hypericaceae*), also known as Saint John’s wort, and its active constituents such as hyperforin and hypericin, have a wide range of medicinal uses, particularly as antidepressant, wound-healing, and antibacterial agents. Clinical studies with *H. perforatum* are divided into three main categories based on the type of disease: psychiatric, endocrine, and skin problems [281,282].

It is well recognized that *H. perforatum* or hyperforin can be used as conventional antidepressants because they inhibit the neuronal uptake of serotonin, norepinephrine dopamine, gamma-aminobutyric acid (GABA) and L-glutamate [282]. In addition, the topical application of *H. perforatum* preparations such as oils or tinctures is used for the treatment of minor wounds and burns, sunburns, abrasions, bruises, contusions, ulcers, myalgia, and many others [283]. In the category of skin problems, it is used for its fibroblasts [284]. In 2D and 3D in vitro dermal constructs, hyperforin reduces the viability of human dermal fibroblasts down to 70% at concentrations of 5–10 µM, by reducing the proliferation of human dermal fibroblasts [285]. Women undergoing surgical childbirth were treated with *H. perforatum* ointment or placebo ointment three times a day for 16 days. The control group remained without any intervention postoperatively. The topical application of *H. perforatum* ointment was safe, and could facilitate cesarean wound healing and minimize the formation of scarring and related pain and pruritus when evaluated on the 40th day postpartum [286].

#### 3.14.6. Loureirin A/B

Loureirin A/B, a major active component of Resina Draconis (Dracaena cochinchinensis)*,* inhibits the proliferation, and suppresses the migration and TGF-β1-induced myofibroblast differentiation, of KD fibroblasts [287,288,289]. Resina Draconis is a type of dragon’s blood resin obtained from *Dracaena cochinchinensis* (Lour.) S.C. chen (Yunnan, China). It has been used as a medicine since ancient times by many cultures. In excision and incision wound models in rats, treatment with an ethanolic extract of Resina Draconis containing loureirin B showed significantly better wound contraction and better skin-breaking strength as compared with the control group [290].

#### 3.14.7. Onion Extract, Contractubex^®^ Gel

Onion extract has been used to treat KD/HTS. Quercetin is the main active compound involved in onions having strong antioxidant and anticancer properties. Quercetin inhibits fibroblast proliferation, collagen production, and the contraction of KD and HTS-derived fibroblasts, and its blocks the signal transduction of IGF-I and the TGF-beta/Smad-signaling pathway in KD fibroblasts [291]. Contractubex^®^ gel, a commercially available topical preparation, is composed of 10% onion extract, 50/U of sodium heparin per 1 g of gel and 1% allantoin [292].

Patients with HTSs or KDs were treated with either onion extract alone (Group 1), silicone gel sheet alone (Group 2), or a combination of onion extract and silicone gel sheet (group 3) for 6 months. A combination of onion extract with an occlusive silicon dressing was thought to be effective in achieving a satisfying decrease in scar height [293]. The efficacy of silicone gel containing either aloe vera or onion extract in preventing postoperative KDs and HTSs was compared in patients who had undergone surgery. Both silicone gel sheets containing aloe vera or onion extract were effective in preventing postoperative scars [262]. The pullulan-based gel containing 5% onion extract and 5% HyA (Treatment-1) or silicone gel alone (Treatment-2) was applied topically on the new post-surgical wounds. Treatment-1 significantly decreased the VSS score, POSAS score, itching, and redness in patients, and manifested a clear reduction in the local inflammation, which might lead to a reduced probability of developing HTS or KD [294]. Patients who received thoracic surgery were treated topically with Contractubex^®^ gel. When evaluated by scar size, the pigmentation of the scar and recurrence rates of HTSs or KDs, Contractubex^®^ gel was useful in scar treatment after thoracic surgery [292]. In rats, skin biopsies were taken to develop full-thickness wounds, and Contractubex^®^ gel, heparin alone, or allantoin alone were topically applied daily after 10 days. Immunohistochemical and ultrastructural observations demonstrated that the Contractubex^®^ gel significantly improved the quality of wound healing and the reduction in scar formation as compared with heparin monotherapy and allantoin monotherapy [295]. Tattoos were removed by using a laser, and the scar area was treated with Contractubex^®^ gel. The local experience revealed that nearly 25% of the patients developed scarring (KD or HTS). The Contractubex^®^ gel group had a statistically significantly lower rate of scarring than the control group [296]. Patients with HTS were treated either with Contractubex^®^ gel or corticosteroid topically, in which the local administration of Contractubex^®^ gel was significantly more effective than corticosteroid treatment. Also, the Contractubex^®^ gel treatment was associated with significantly fewer adverse events than topical corticosteroid application [297,298]. The efficacy of onion extract in the management of abdominal HTS formation was examined using Contractubex^®^ gel in patients. Better results for vascularity, pigmentation and height subscales of the VSS after the surgical removal of the primary caesarean scar were obtained with Contractubex^®^ gel [299]. In contrast, however, some research groups reported the following: the efficacies of silicone gel, silicone gel sheeting and Contractubex^®^ gel for the treatment of post-burn HTSs was compared. Silicone products, either in gel or in sheet, were found to be superior to Contractubex^®^ gel in the treatment of HTSs [300]. Separately, vascularity, pliability, pigmentation and height, as subjective scar parameters, were not statistically different in post-upper-extremity sharp injury wound HTSs among silicone gel, Contractubex^®^ gel, and no intervention groups [301].

#### 3.14.8. Resveratrol

Resveratrol is the most well-known polyphenolic stilbenoid, present in grapes, blueberries, mulberries, peanuts, raspberries, rhubarb, and several other plants. The effects of resveratrol on the immune system are associated with widespread health benefits for different autoimmune and chronic inflammatory diseases [302]. Resveratrol significantly inhibits cell growth by arresting the cell cycle at the G1 phase and inducing apoptosis in the fibroblasts; it also decreases hydroxyproline (or collagen) levels, and downregulates the expression levels of type I and III procollagen mRNA in human HTS fibroblasts [303]. In an in vitro study, the treatment of KD fibroblasts with resveratrol decreased type I collagen, α-SMA, and HSP47 expression in a dose-dependent manner. In addition, resveratrol diminishes TGF-β1 production and suppresses their proliferation, and it induces apoptosis of the KD fibroblasts, without any adverse effects on normal skin fibroblasts [304]. Hypoxia promotes proliferation and inhibits the apoptosis of KD fibroblasts, but resveratrol can reverse the effect of hypoxia on KDs, inhibit collagen synthesis in KD fibroblasts, and promote cell apoptosis through the downregulation of hypoxia-inducible factor (HIF)-1α [305].

#### 3.14.9. Saireito (or Sairei-to)

Saireito (or Sairei-to), the traditional Japanese herbal (Kampo) medicine, is used in clinic to treat KD/HTS as an orally administered drug. Saireito extract granules (Kracie Saireito Extract Granules^®^) are commercially available. The mechanism of saireito’s inhibitory activity on fibroblast cells is reported to be due to the suppression of TGF-β1-induced Smad2/3 phosphorylation [254,306,307]. The clinical efficacy of saireito in treating KDs/HTSs was compared with tranilast (Rizaben^®^ capsule 100 mg) in patients with KDs or HTSs after burns, trauma or surgery, in which the dose of saireito was 2.3 g applied three times per day for 12 weeks and that of tranilast was 100 mg three times per day for 12 weeks. The saireito and tranilast groups showed high rates of improvement, at 54.3% and 47.5%, respectively. The early improvement in symptoms such as blushing, induration, and swelling was more significant in the saireito group than in the tranilast group [308].

#### 3.14.10. Shikonin

Shikonin, one of the active components of traditional Chinese herbal medicines such as the dried root of Zicao, exhibits antiproliferative, anti-inflammatory, and anti-angiogenic activities. Shikonin has a role in inducing ROS, suppressing the release of exosomes, and inducing apoptosis [309]. Shikonin inhibits the expression of p63 (a type II integral membrane protein), cytokeratin 10, α-SMA, TGF-β1, and collagen I, which play important roles in HTS formation, suggesting that shikonin has potential utility as a novel scar therapy [310]. Also, shikonin promotes HTS repair via the autophagy of HTS-derived fibroblasts, where the potential mechanism may be related to the AMP-activated protein kinase/mammalian target of rapamycin (mTOR) signal pathway [311]. In the human skin fibroblasts in vitro, shikonin reduced TGF-β1-induced collagen production through the extracellular-signal-regulated kinase/Smad signaling pathway and attenuated TGF-β1-induced cell contraction by downregulating α-SMA expression [312].

Burn skin exhibits evidence of Warburg-like metabolism, as in KDs. Targeting this altered metabolism could change the trajectory toward normal scarring, indicating the clinical possibility of using shikonin for abnormal scar prevention [313]. The dissolvable shikonin-HyA microneedles were developed to enhance skin penetration. In an in vitro study using HTS fibroblasts, the delivery system significantly reduced the viability and proliferation of the HTS fibroblasts and downregulated the fibrotic-related genes (i.e., TGFβ1, FAP-α and COL1A1) [314].

#### 3.14.11. Emodin

Emodin is a major component of the widely used Chinese herb rhubarb (rheum rhaponiticum), and has been used to treat inflammation in several types of disease. The ethyl acetate extract of rhubarb (rheum rhaponiticum) contains emodin (a natural anthraquinone derivative), rhein and gallic acid as active compounds, and shows anti-proliferative activity on HTS fibroblasts. Emodin significantly attenuated HTS inflammation in a mouse wound model, as evaluated by the scar elevation index, collagen structure and inflammation [315,316]. In HTSs on rabbit ears, emodin gel significantly decreased the hardness, and the expressions of TGF-β and IL-1, in HTSs compared to the non-treatment control group. Also, the emodin gel decreased the hardness of HTSs and inhibited the proliferation of fibroblasts in the local area [317]. In addition, emodin can exert its anti-fibrotic effect via the suppression of TGF-β1 signaling and the subsequent inhibition of inflammation, HSP47 expression, myofibroblast differentiation and ECM deposition in rats [318]. It was also reported that emodin attenuates HTS formation and fibrosis by suppressing macrophage polarization in a rat wound model, which is associated with the inhibition of the neurogenic locus notch homolog (Notch) and TGF-β pathways in macrophages [299].

#### 3.14.12. Other Plant-Based Medicines (Glabridin, Kaempferol, Tripterine, Wubeizi)

It is reported that glabridin, a typical flavonoid isolated from the Glycyrrhiza glabra, can block focal adhesion kinase (FAK)–steroid receptor coactivator (Src) complex formation in cancers; it also exhibits therapeutic effects on HTS pathology, probably through the co-deactivation of FAK/Src, which further results in FAK-Src de-association [319]. Also, glabridin can suppress the human KD fibroblast cells’ proliferation by inducing apoptosis and reducing collagen production [320].

Kaempferol, also known as kaempferol-3 or kaempferide, is a flavonoid compound that naturally occurs in tea, as well as numerous common vegetables and fruits, including beans, broccoli, cabbage, grapes, strawberries, tomatoes, citrus fruits, apples and grapefruits [321]. Kaempferol inhibits fibroblast collagen synthesis, proliferation and activation in hypertrophic scars via targeting the TGF-β receptor type I in HTS [322].

Tripterine, active in Thunder God Vine (Tripterygium wilfordii), a traditional Chinese medicine, ameliorates the pathological characteristics of KD fibroblasts that are associated with KD formation and growth by inducing *ROS* generation and activating the c-Jun N-terminal kinases (JNK) signaling pathway [323]. Also, tripterine, a bioactive pentacyclic triterpenoid compound, inhibits the growth of NIH/3T3 cells by decreasing the expressions of MMPs, VEGF and basic FGF in an in-vitro study using NIH/3T3 cells [324].

Wubeizi ointment aqueous solution restricts KD fibroblast proliferation by downregulating the expressions of type I and III procollagen and therefore reducing collagen deposition in KD tissues in a study using KD-derived fibroblast [325]. Wuweizi is a traditional Chinese medicine known as an astringent drug. In a KD mouse model and in human KD-derived fibroblasts, Wubeizi ointment suppressed KD formation through the inhibition of fibroblast proliferation and the promotion of fibroblast apoptosis [326]. In an in vitro study using KD-derived fibroblasts, the Wubeizi ointment inhibited the proliferation of the KD-derived fibroblasts in a time- and dose-dependent manner [327].

### 3.15. Statins (Simvastatin, Lovastatin, Pravastatin, Atorvastatin)

Statins such as simvastatin, lovastatin, pravastatin, and atorvastatin are widely prescribed cholesterol-lowering drugs used for hypercholesterolemia all over the world. In addition to their lipid-lowering effect, statins are also known for their pleiotropic and anti-inflammatory activity, because statins can alleviate tissue fibrosis originating from a variety of pathological insults [328,329]. In Caucasian patients, HTSs form at a rate of 29.7% after major cardiac surgery with median sternotomy and cardiopulmonary bypass. Logistic regression analysis confirmed the protective role of statins after adjustment for age, in which higher doses of statins showed a more intensive protective effect. Thus, statin use could be effective in preventing the recurrence of HTSs after cardiac surgery through median sternotomy [328].

#### 3.15.1. Simvastatin

In an in vitro study using KD fibroblasts, simvastatin, a 3-hydroxy-3-methylglutaryl coenzyme-A reductase inhibitor that is used to reduce cholesterol levels, inhibited the TGF-β1-induced production of type I collagen, CTGF, and α-SMA [330]. The effects of simvastatin on the proliferation, apoptosis and protein expressions of KD fibroblasts were examined under normoxia, hypoxia or TGF-β1 treatment. Simvastatin significantly induced the apoptosis of KD fibroblasts, decreased type I collagen and CTGF, and increased Tissue Inhibitor of Metalloproteinase (TIMP)-1 under hypoxia, in which there was no significant effect under normoxia for 48 h. Also, simvastatin significantly inhibited the expression of CTGF under TGF-β1 treatment [331]. In a rabbit HTS model, topical cream formulations of simvastatin and pravastatin were prepared, and their abilities to reduce scar hypertrophy and the attenuation of dermal fibrosis were evaluated. The topical application of 10% simvastatin cream alone, but not 2% simvastatin and 10% pravastatin creams, significantly attenuated the hypertrophy of resultant scars compared with vehicle cream alone. Data indicate that topical 10% simvastatin cream antagonizes dermal fibrosis and reduces hypertrophy [332].

#### 3.15.2. Atorvastatin, Lovastatin, Pravastatin

The usefulness of atorvastatin for the treatment of HTS, as well as those of 5-FU and TAC, was reported using an animal-free human tissue-engineered HTS model [333]. In human Tenon’s capsule fibroblasts, atorvastatin inhibited proliferation and migration, and induced cell apoptosis. Also, atorvastatin downregulated the expression level of TGF-β2, and protein levels of SMA, p38, Smad3, fibronectin, collagen I, and collagen III [334]. In a rabbit ear HTS model, treatment was performed with intralesional simvastatin, lovastatin, or pravastatin at low, medium, or high doses on post-wounding days 15, 20, and 25. Low-dose (40 μM) simvastatin, lovastatin, and pravastatin each significantly reduced scar elevation by 21.9%, 25.8%, and 22.8%, respectively, and low-dose simvastatin demonstrated a significant reduction in CTGF expression. In contrast, medium—(120 μM) and high-dose (400 μM) statin groups did not change the scar elevation significantly [335]. In a rabbit ear model, topical treatment (day 6–28 post-operation) with liposomal simvastatin and pravastatin at 6.5% concentrations significantly reduced scar elevation index, also decreased type I/III collagen content and myofibroblast persistence, in the wound. Also, liposomal pravastatin treatment decreased expression levels of transcripts encoding CTGF, collagen I, and collagen III in scar tissue [336].

### 3.16. Steroids (Triamcinolone acetonide, Dexamethasone, Hydrocortisone Acetate, Methylprednisolone)

Glucocorticoids and their derivatives such as TAC, dexamethasone (DEX), hydrocortisone acetate, and methylprednisolone are widely used for the treatment of inflammatory diseases, autoimmune diseases, and cancer. Glucocorticoids also exhibit broad and potent physiological and therapeutic effects. Their important action is the modulation of gene transcription through many distinct and complementary mechanisms, and target genes include most inflammatory mediators such as chemokines, cytokines, growth factors and their receptors. However, their clinical uses are limited due to the induction of side effects, such as dermatologic and immunological side effects, as well as glucocorticoid resistance [337,338].

#### 3.16.1. Triamcinolone Acetonide (TAC)

It is reported by many researchers that intralesional TAC injection has remained a gold standard in non-surgical management [89,93,94]. TAC is a synthetic glucocorticoid (class of corticosteroids) exhibiting antiallergic activity, and in clinic, this drug is injected into various locally affected sites, for example, in intra-articular, intra-soft tissue, intra-synovial, local intradermal, intranasal, ear canal, esophagal sites, and so on, in addition to oral, intravenous, intramuscular, and inhalation administration. In the case of skin diseases such as KDs and HTSs with raised skin scars, intralesional administration is the most widely used and effective treatment modality [339]. In an in vitro study, TAC significantly increased the production of basic FGF and decreased the production of TGF-β1 by human KD fibroblasts in a serum-free in vitro model [340]. TAC downregulates pro-fibrotic genes and ECM regulators, such as TGF-β, collagens and integrins, in idiopathic carpal tunnel syndrome [341]. In addition, TAC reduced the proteins and mRNA expression levels of COL1, COL3, and α-SMA, and suppressed the proliferation, invasion, and migration of human HTS fibroblast in a dose-dependent manner, and the intralesional injection of TAC significantly reduced the proportion of scars in mice with scar tissue [342]. In nude mice implanted with human KDs, intralesional TAC significantly increased apoptosis in the KDs [343]. The pharmacological potency of glucocorticoids shows dose-dependency, with a greater potency is observed at a higher dose (concentration), although side effects are also induced [342]. The current standard of care for KD is intralesional steroids such as TAC [6]. In KD scars, the levels of pro-α1(I) type I collagen mRNA in the dermis are greatly elevated. Intralesional TAC injection immediately after KD excision decreases pro-α1(I) collagen transcripts, compared with untreated skin. In the TA-treated skin, the collagen bundles were thinner and less dense [344]. The clinical efficacy in managing HTSs and KDs was compared between intralesional TAC and VER. Both drugs reduced the vascularity, pliability, height and width of the scar after 3 weeks and one year of follow-up, but not the pigmentation and length of the scars. Adverse drug reactions were more severe with TAC than with VER [93]. Patients with KD were treated with intralesional TAC once a week for 4 weeks. After the last treatment, the dermal tissue layer thickness decreased to 39.0% as compared to before treatment [345]. The clinical outcomes of intralesional excision followed by postoperative intralesional TAC injection for auricular KD treatments were evaluated. The recurrence rate was 5% within the 24-month follow-up period, and a significant reduction in height and volume was achieved in 95% of patients. This low recurrence rate is comparable with that of postoperative radiation therapy [346]. The efficacy of intralesional TAC injection in KD management was evaluated using an Antera3D^®^ imaging system, and a reduction in KD dimensions and symptoms such as itching and pain was observed, even if the treatment had local minimal adverse effects [347]. It was reported that the surgical excision followed by sub-dermal TAC injection immediately after excision offered the safe, long-lasting and cost-effective management of caesarean KD scars and the prevention of KD recurrence [348].

#### 3.16.2. Dexamethasone (DEX)

DEX, a synthetic glucocorticoid that binds to the human glucocorticoid receptor (GR) and is one of the most effective anti-inflammatory glucocorticoids, induces KD regression via interaction with the GR, and suppresses endogenous VEGF expression and fibroblast proliferation in primary KD fibroblast cultures [349]. Also, DEX significantly reduced the KD volume and cellularity, and induced epidermal shrinkage, in a long-term organ culture of KD in diseased tissue [350]. DEX and green tea polyphenols (GTP) were incorporated into electrospun polymer ultrafine fiber meshes as a co-delivery system, where the efficacy of KD treatment as a surgical dressing was evaluated in nude mice implanted with human KD tissues. Histological analysis after 3-month treatment showed that the DEX/GTP-loaded fiber meshes significantly induced the degradation of collagen fiber in KD [351]. The effectiveness of glucocorticoids in preventing HTSs in burn patients was compared among hydrocortisone, methylprednisolone, DEX, TAC, and prednisone. In patients with a 0–19% total body surface area (TBSA) burn, methylprednisolone led to a decreased risk of developing HTS diagnosis. Methylprednisolone was associated with reduced HTS diagnosis in burn patients independent of TBSA. In those with a 20–39% TBSA burn or 40–100% TBSA burn, prednisolone showed an increased risk of developing an HTS diagnosis. Methylprednisolone was associated with reduced HTS diagnosis in burn patients independent of TBSA. In those with a 20–39% TBSA burn or 40–100% TBSA burn, DEX showed an increased risk of developing an HTS diagnosis [352].

### 3.17. Regenerative Medicine (Fat Grafting, Platelet-Rich Plasma, Adipose-Derived Stroma Vascu-Lar Fraction, Adipose-Derived Stem Cells, Mesenchymal Stem Cells, Hyaluronic Acid)

Wound healing is a complex process that depends on the presence of various types of cells, growth factors, cytokines, and the elements of ECM. Tissue regeneration technology remarkably enhances skin repair via re-epidermalization, epidermal–stromal cell interactions, angiogenesis, and the inhabitation of hypertrophic scars and keloids [353]. The important cells involved include epidermal stem cells, dermal precursors of fibroblasts, adipose-derived stem cells (ASC), and bone marrow cells. The activities of these cells are strictly regulated by various growth factors, such as epidermal growth factor (EGF), fibroblast growth factor (FGF), PDGF, TGF, and IL [354]. These indicate that biocomponents involving cells, fractions and growth factors are closely related to wound healing. In in vitro primary dermal fibroblasts isolated from cultures of HTSs, the expression of TGF-β1 mRNA in the fibroblasts cocultured with platelet-rich plasma (PRP) was significantly lower than in those of platelet-poor plasma (PPP) treatment from 4 to 13 days of culture. The connective tissue growth factor (CTGF) levels and mRNA expression in the PRP groups were lower than those in the PPP groups [355]. Thus, autologous platelet-based concentrates represent increasingly popular adjuncts to a variety of medical, surgical and aesthetic interventions. Their beneficial potential rests on the ability to deliver a high concentration of growth factors to the target tissues [356]. In addition, platelets are important donors of mitochondria, and platelet-derived mitochondria can promote wound healing by reducing apoptosis caused by oxidative stress in vascular endothelial cells [357]. The efficiency of various regenerative medicine approaches, such as platelet-rich plasma, cell therapy, stromal vascular fraction, exosomes and stem cell-conditioned medium, are continually emerging with a focus on personalized, patient-specific treatments, and it is reported that regenerative medicine is an effective method with minimal side effects that can be used in the treatment of HTSs and KDs [353,358,359,360].

#### 3.17.1. Fat Grafting

In scar treatment, an injection of fat graft (adipose tissue) is performed. Adipose tissue is a connective tissue that contains a reserve of mesenchymal stem cells, and this treatment can reduce pain and increase scar elasticity [361,362]. Fat grafting accelerates revascularization and decreases fibrosis in mice with thermal injury [363]. Autologous fat grafts have many clinical applications in breast surgery, facial rejuvenation, buttock augmentation, and Romberg syndrome, as well as in the treatment of liposuction sequelae, indicating autologous fat grafting is a good method for the correction of scars on the face instead of traditional scar surgical excisions [362]. Adipose tissue can be safely grafted into scars to improve refractory neuropathic pain after severe scarring, such as via burn injury [364,365,366,367]. Based on these results, it was considered that the treatment of severe scars such as burn injuries with fat grafting can prevent the induction of KDs and/or HTSs at injury sites [368,369].

#### 3.17.2. Platelet-Rich Plasma (PRP)

Autologous PRP is an effective adjuvant in managing skin graft donor sites. PRP can reduce pain and pruritus and improve wound healing of the skin graft donor site [370]. When PRP was injected intraoperatively into KD patients who did not respond to cortisone injection or radiotherapy, KD scars were completely resolved in 53% of patients and completely relapsed at 2 years in 29% of patients. The mean VSS score improved to 3.82 ± 1.98 from 8.18 ± 2.38 at 2 years, indicating PRP injection is an effective and safe method when used as an adjunctive therapy to resection for treating KD scars refractory to conventional therapy [371]. When KD scars were treated with either intralesional botulinum toxin type-A (BTX-A), PRP, or TAC, both BTX-A and PRP could yield a chance for cosmetically better outcomes in KD treatment than conventional TAC injection [372]. However, it was also reported that PRP significantly reduces postoperative recovery time, but does not improve patient outcomes when looking at skin elasticity, improvement of the nasolabial fold, or patient satisfaction [373]. Patients with KDs were treated with either intralesional triamcinolone, verapamil (VER), 5-fluorouracil (5-FU), or PRP. When evaluated by the Patient and Observer Scar Assessment (POSAS, evaluation of pain, itching, pigmentation, stiffness, thickness, flatness of scar, vascularization, pliability, relief, etc.) score, their efficacies were as follows: VER > triamcinolone = PRP > 5-FU [374].

#### 3.17.3. Adipose-Derived Stromal Vascular Fraction (SVF)

Both adipose-derived stromal vascular fraction (SVF) and mesenchymal stem cells (MSC) can significantly reduce the clinical and histological parameters of HTS, although MSC is more efficient than SVF in remodeling HTS [375]. In the patients treated with SVF-enhanced autologous fat grafts, a 63% maintenance of contour restoration was observed after 1 year, although it was only 39% in the control group treated with centrifuged fat graft [362]. The efficacy when used for HTS treatment was compared between BTX-A and SVF gel in a rabbit ear model, in which BTX-A showed better anti-scar efficacy than SVF-gel [376]. In a humanized skin graft model in nude mice, the therapeutic effect in remodeling HTS was compared between SVF and human adipose tissue-derived MSC. Both SVF and MSC significantly reduced collagen contents and dermis thickness in the skins of treated mice, but MSC appeared to be more efficient than SVF [375]. In intermediate–deep acute burns, quicker healing was observed in the case area after treatment with SVF and a scaffold of HyA, as opposed to the control one, which eventually underwent skin grafting. Both areas showed a tendency towards HTS development, while the patient satisfaction Visual Analogue Scale (VAS, one of the pain rating scales) was 7 on the case side and 2 on the control side [377]. Patients with KD were treated with either SVF or TAC (control). No adverse events were observed in the SVF treatment group, while ulceration (grade II adverse event) was observed in a single patient in the TAC treatment group. It was concluded that an autologous adipose-derived SVF is feasible and safe for use in the treatment of KD in low- and middle-income countries [378].

#### 3.17.4. Adipose-Derived Stem Cells (ASC)

Stem cells are a population of undifferentiated cells characterized by the ability to extensively proliferate (self-renewal); they usually arise from a single cell (clonal), and differentiate into different types of cells and tissue (potent) [379]. A peptide derived from a conditioned medium of ASCs inhibits collagen, and ACTA2, or smooth muscle actin (SMA) mRNAs, in HTS fibroblasts facilitate wound healing and attenuate collagen deposition by binding with the pyruvate carboxylase (PC) protein, which inhibits PC protein expression in a mouse model [380]. Treatment with ASC strikingly elevated cyclooxygenase-2 (COX-2) mRNA and protein expressions in KD dermis-derived fibroblasts, which would play a crucial role in mediating the ASC-conditioned medium-induced apoptosis and anti-proliferation effects. In the nude mouse model, expressions of arachidonic acid, COX-2 and prostaglandin E2 (PGE2) were higher in the translated KD tissues after ASC-conditioned medium injection than in the controls [381]. When KD fibroblasts were cultured with ASC-exosomes, the proliferation rate, migration rate, collagen synthesis levels, relative mRNA and protein expression levels of α-SMA, TGF-β1, and Smad3 of KD fibroblasts were decreased, and the apoptosis rate was increased in a manner dependent on the concentration of ASC-exosomes [382,383]. In a rabbit model, a culture medium containing a human ASC extracellular vesicle (0.1 mL) was injected into a wound made in the ear during wound healing. The treatment prevented the formation of HTSs on postoperative day 28, and suppressed collagen deposition and myofibroblast aggregation, as compared with the medium alone [384]. ASC emerged as a potential solution for alleviating HTS [385].

#### 3.17.5. Mesenchymal Stem Cells (MSC)

Mesenchymal stem cells (MSCs) secrete diverse growth factors and cytokines, induce angiogenesis, reduce inflammation, and promote fibroblast migration and collagen production [386]. Bone marrow-derived MSCs attenuate the proliferative and profibrotic phenotype associated with KD and HTS fibroblasts, and inhibit ECM synthesis through a paracrine signaling mechanism [387]. The most investigated types of MSCs are those isolated from the human umbilical cord blood, adipose tissue and bone marrow [388]. Both SVF and human adipose tissue-derived MSCs significantly reduce the clinical and histological parameters of HS, and human MSCs are more efficient than SVF. MSCs are a valuable cell source in regenerative medicine, and the conditioned medium obtained from MSCs reportedly inhibits inflammation. For example, conditioned medium obtained from amnion-derived MSCs significantly suppresses the proliferation of KD fibroblasts, as well as the TGF-β-induced upregulation of α-SMA in KD and normal fibroblasts and collagen I in KD fibroblasts [389]. Human MSC-conditioned medium inhibits the proliferation and collagen synthesis of human KD-derived fibroblasts, and reduces inflammation and fibrosis in the KD implantation model [390]. When the treatment efficacy of intralesional umbilical cord-derived MSC (UC-MSC) in relation to KD therapy was evaluated by the decrease in KD volume, symptoms, and type 1:3 collagen ratio, as well as the increasing levels of IL-10, the intralesional injection of UC-MSC and conditioned medium of UC-MSC were more effective than intralesional TCA [391].

The therapeutic effect of MSCs is attributed to the higher expression of TGF-β3 and hepatocyte growth factor (HGF), which are important anti-fibrotic mediators, and to higher levels of matrix MMP-2 and MMP-2/tissue inhibitor of TIMP-2 ratio, which reflect the remodeling activity responsible for fibrosis resorption [375]. Exosomes derived from human adipose MSC attenuate the deposition of collagen, the trans-differentiation of fibroblasts-to-myofibroblasts, and the formation of HTS by in vitro and in vivo experiments [392]. In clinic, both bone marrow and UC-MSCs effectively improved burn injury healing, as evaluated by late complications of HTSs, and the hypopigmentation and hyperpigmentation of scars, in which the recurrence rate of HTSs was 40% in patients treated with conventional early excision and graft, 15% in patients treated with bone marrow MSCs and 20% in patients treated with UC-MSCs [393]. The effectiveness of MSC therapy in the treatment of KDs and HTSs was evaluated by addressing macroscopic and histological appearances and immunohistochemistry. Improvements in outcomes were observed in all cases with MSCs or MSC-conditioned media treatments, without complications [394]. Autologous adipose tissue-derived MSCs were applied to treat post-burn scars. When evaluated by the VSS score, the average VSS score decreased to 2.34 (ranging from 1 to 4) points 6 months after the surgical procedure, from 7.88 (ranging from 4 to 11) points before treatment [395]. When the clinical efficacy of the UC-MSC and the conditioned medium of MSCs were evaluated by the reduction in KD volume and symptoms, type 1:3 collagen ratio, and the increased levels of IL-10, the intralesional injections of these MSCs were more effective than TAC in KD therapy [391]. In a review article, MSC-conditioned media was tested intravenously, intraperitoneally, subcutaneously, intradermally, intralesionally or topically. MSC-conditioned media could improve wound healing, hair restoration, skin rejuvenation, atopic dermatitis, and psoriasis in both animals and humans [396,397].

#### 3.17.6. Hyaluronic Acid (HyA)

Hyaluronic acid (HyA), one of the main components of the ECM, is a glycosaminoglycan composed of alternating N-acetyl-D-glucosamine and D-glucuronic acid moieties. HyA is a ubiquitous component of connective tissue, where it forms a matrix and plays an important role in the maintenance of matrix structure and water balance [339]. In addition, HyA plays a multifaceted role in regulating various biological processes, such as skin repair, diagnosis of cancer, wound healing, tissue regeneration, anti-inflammatory actions, and immunomodulation, and HyA has been employed as one of the imperative components of cosmetic and nutricosmetic products [398]. In the tissue regeneration process, HyA is considered one of the key players. HyA can modulate inflammation, cellular migration, and angiogenesis, the main phases of wound healing, via specific HyA receptors [399]. The administration of exogenous HyA via liquid jet injection is reported to be a beneficial therapy for dermatology conditions [400]. In an in vitro study, HyA decreases proliferation activity, pro-collagen I expression, TGF-β1 expression, and TGF-β1 release in KD fibroblast cultures [401]. In clinic, the combined treatment of HyA with cortisone showed a complete resolution of the KD without recurrence several months later in a KD patient [402]. Also, a combination of bleomycin (BLM) and HyA permitted the delivery of BLM without pain, a fast onset of action and good bioavailability, and it inhibited the proliferation of human HS fibroblasts and the secretion of TGF-β1 in vitro [403]. Patients with multiple sternal KDs who received monthly steroid injections received an additional injection of BTX-A/HyA on the same day as the first injection of the steroid. The microneedle delivery of BTX-A/HyA gave higher patient satisfaction when evaluated by VSS and VAS scores [404]. In a review article, HyA was reported to be an effective carrier for both topical and transdermal deliveries due to its unique viscoelasticity, biocompatibility, biodegradability, non-immunogenicity, and biomedical benefits for the skin [405].

### 3.18. Comparison of Clinical Efficacy between Triamcinolone Acetonide and Other Drugs

The clinical efficacy in treating HTS was compared among intralesional TAC, 5-FU and BTX-A in a rabbit ear HTS model. TAC and 5-FU were similarly effective compared to monotherapy, but BTX-A was not effective in established HTSs [406]. The clinical efficacy in treating KDs was compared between intralesional TAC and 5-FU. The patients treated with 5-FU experienced side effects such as hyperpigmentation, pain at the injection site (95% of patients), and superficial ulceration. Thus, TAC appeared to be a better tolerated and less toxic alternative to 5-FU in the management of KD [407]. In contrast, there was no statistically significant difference in the remission rate at 6 months between the TAC and 5-FU groups (60% vs. 46%, respectively) in patients with KD. Local adverse effects such as atrophy and telangiectasia were significantly higher in the TAC group compared to the 5-FU group [408]. The efficacy of intralesional TAC was compared with VER in treating HTSs and KDs. VER deserves better positioning in the wide armamentarium against HTSs, because of its low cost and fewer adverse effects [89]. In addition, it was also reported that in treating KD/HTS, intralesional VER is extremely low-cost and has fewer adverse effects, thus grounding several therapeutic possibilities to alternate with TAC or be used simultaneously in larger (or multiple) scars, compared to intralesional TAC [94]. The efficacy in the prevention of KD recurrence after excision was compared between intralesional TAC (10 mg/mL) and VER (2.5 mg/mL), which patients received at monthly intervals (four doses). VER was safe but not as effective as TAC in preventing KD recurrence after excision [409]. Similarly, the clinical efficacy of intralesional TAC was compared with that of intralesional VER in the treatment of KDs. Better improvements in height and pliability were observed resulting from TAC in comparison with VER [410]. The efficacy and safety were compared between TAC and VER for KD and HTS treatments. Between them, there was no significant difference in the reduction in height, vascularity, pliability, and degree of pigmentation. It was concluded that VER might be used as an alternative treatment when TAC results in adverse outcomes [91]. The clinical efficacy of intralesional TAC injection in KD treatment was compared with that of intralesional 5-FU, VER and PRP. When efficacy was evaluated by POSAS, VER was the most effective, and PRP was as effective as TAC with no serious side effects. 5-FU was less effective in treating the KD [374]. Other reports are as follows: the rates of KD recurrence after surgical excision alone and postoperative injection with TAC or INT-α2b were 51.1% (control), 58.4%, and 18.7%, respectively, indicating that postoperative TAC injections do not reduce the number of KD recurrences efficiently [411]. The clinical efficacy in treating KD was compared between intralesional TAC and BLM. BLM was more efficacious than TAC when the efficacy was evaluated by POSAS at 24 weeks follow-up [412]. In a review article, the treatment efficacies of intralesional TAC and BTX-A injection for HTS and KD treatment were compared. When evaluated by VAS, BTX-A injection was more effective than an injection of intralesional corticosteroid or placebo [413]. Clinical efficacy for the treatment of KD/HTS was compared between intralesional TAC and enalapril. The evaluation was made by VSS and POSAS. Both TAC and enalapril showed the same clinical effects, indicating that enalapril could be a safe alternative to steroids in the treatment of KD and HTS [414].

**Table 2:** Drugs used for keloids/hypertrophic scars and their pharmacological action.

Drugs	Pharmacological Action
ACE inhibitor Captopril Enalapril Losartan	Angiotensin-converting enzyme (ACE) inhibitors reduce fibroblast proliferation, suppress collagen and TGF-β1 expression, and downregulate the phosphorylation of SMAD2/3 and TAK1. ACE inhibitors such as captopril, enalapril, and losartan inhibit the production of angiotensin II, TGF-β1 and ECMs such as collagen [28,29,30].
Antiallergic agent Tranilast	Tranilast, an orally administered drug, suppresses type I allergic reactions by inhibiting the release of chemical mediators such as histamine and leukotrienes from mast cells and various inflammatory cells. It also inhibits the production of collagen, TGF-β, INF-γ, IL-6, IL-10, IL-17, VEGF, MMP-2, MMP-9, TNF-α, some other angiogenic, and inflammatory factors [38,40,42,43].
Antisense drug TGF-β1 antisense SMAD3 antisense hTERT antisense	Topically applied TGF-β1 antisense preparations downregulate TGF-β1 protein levels and improve scar histology as determined by the scar elevation index in vivo [62]. Treatment with SMAD3 antisense inhibits SMAD3, a primary inducer of fibrosis, and suppresses collagen production in KD fibroblasts [63]. Human telomerase reverse transcriptase (hTERT) antisense oligodeoxynucleotide suppresses the growth and proliferation of KD fibroblasts and inhibits telomerase activity in KD fibroblasts [64].
Antiviral cytokines Interferons	Interferon (IFN)-α, β, and γ suppress collagen synthesis by dermal fibroblasts. IFN-γ also suppresses collagen synthesis by myofibroblasts, synovial fibroblast-like cells, and type II collagen synthesis in human articular chondrocytes [68].
Calcium-channel blockers Verapamil	Verapamil inhibits transmembrane calcium influx, the growth and proliferation of vascular smooth muscle cells and fibroblasts, and the synthesis of ECM proteins (collagen, fibronectin, proteoglycans) [76,78].
Chemotherapeutics Bleomycin Camptothecin 5-Fluorouracil Mitomycin C Paclitaxel Tamoxifen	Chemotherapeutics such as bleomycin, camptothecin and 5-fluorouracil can induce apoptosis, autophagy and cell cycle arrest in tumor cells by inhibiting DNA synthesis and interfering with RNA. Metabolites of mitomycin C also interfere with the synthesis of DNA, RNA and proteins [95,96,97,122]. Liposomal paclitaxel can suppress the production of TNF-α, IL-6 and TGF-β and inhibit the expression of α-SMA and collagen I in human KD fibroblast [133]. Tamoxifen decreases the expression of TGF-β1, with the consequent inhibitions of both fibroblast proliferation and collagen production [135].
Enzyme Collagenase Hyaluronidase	The intralesional injection of collagenase can degrade collagen fiber directly and decrease KD volume promptly [145]. Hyaluronidase produces low-molecular-weight fragments during the digestion of high-molecular-weight hyaluronic acid. These fragments are known to stimulate angiogenesis and activate mesenchymal stem cells [144].
Fat-soluble vitamin Vitamin A Vitamin D3 Vitamin E	Vitamins are effective in the treatment of inflammatory dermatoses, acne, pigmentation disorders and wound healing [153]. Vitamin A significantly reduces fibroblast proliferation and collagen synthesis in vitro and in vivo [158]. Vitamin D3 slows the progression of tissue fibrosis by KD fibroblasts and inhibits collagen synthesis in dermal fibrosis [170,173]. Vitamin E supplementation is beneficial for wound repair and immune functions [176].
Immunomodulator Tacrolimus Imiquimod	Tacrolimus inhibits KD fibroblast proliferation, migration and collagen production enhanced by TGF-β1. The increase in TGF-β receptor I and II expression in TGF-β1-treated KD fibroblasts is suppressed by tacrolimus treatment [179]. It also suppresses smooth muscle actin, reduces mucin, and improves the quality of collagen fibers and the density of elastic fibers [183]. Imiquimod and its metabolite, immune-modulators, induce IFN-α in human blood cells, and IL-1, IL-6, IL-8, and TNF-α in human PBMC cultures in vitro [185,186]
Monoclonal antibody Dupilumab Anti-TGF-β1 antibody Anti-VEGF-A antibody	Dupilumab, a human monoclonal IgG4 antibody, inhibits IL-4 and IL-13 signaling by binding to the IL-4Rα receptor subunit affecting cellular transcription [195,196,197,198,199]. TGF-β has differential temporal effects in the healing of the wound, and anti-TGF-β1 antibodies can modify the healing process [201,202]. VEGF-A is a key cytokine in developing blood vessels in normal tissues and other tissues undergoing abnormal angiogenesis. Anti-VEGF antibodies exhibit therapeutic utility in blocking VEGF-induced angiogenesis [207].
Neurotoxin Botulinum toxin A	Botulinum toxin A is a potent neurotoxin protein that exerts its effect at the neuromuscular junction by inhibiting the release of acetylcholine, which causes temporary chemical denervation, induces temporary muscular paralysis, and relieves the tension on wound edges [202,203,204,205,206,207,208,209].
Peripheral vasodilator Pentoxifylline	Pentoxifylline regulates TGF-β1-induced fibroblast activation, modifies the expression of collagen types I and III by human fibroblasts, and inhibits the proliferation and rate of collagen synthesis of fibroblasts [230,415,416].
Photosensitizer prodrug 5-Aminolevulinic acid	Photodynamic therapy using topical 5-aminolevulinic acid and red light can reduce cell viability and TGF-β1-mediated signaling by inducing cell apoptosis in human HTS fibroblasts [246].
Plant-based Aloe vera Centella asiatica Contractubex Curcuminoids (curcumin) Green tea (EGCG), Hyperforin Loureirin A/B Onion extract (quercetin) Resveratrol Saireito Shikonin Emodin Glabridin Kaempferol Tripterine Wubeizi	Aloe vera exhibits potential antioxidant, anti-inflammatory, and wound-healing activities [257,263]. Centella asiatica decreases fibroblast proliferation, inhibits type I and type III collagen protein and mRNA expressions, and reduces the expression of both TGF-βRI and TGF-βRII at the transcriptional and translational level [265,268]. Contractubex has a softening and smoothing effect and improves the quality of wound healing by reducing scar formation [295,297]. Curcumin blocks the elevation of ECM and TGF-β1/p-SMAD-2 levels in a dose-dependent manner in KD fibroblasts [273]. EGCG can downregulate the expression levels of collagen by inhibiting the TGF-β/Smad3 signaling pathway [278,280]. Hyperforin reduces the viability of human dermal fibroblasts by reducing the proliferation of human dermal fibroblasts [285]. Loureirin A/B inhibits the proliferation, migration and TGF-β1-induced myofibroblast differentiation of KD fibroblasts [287,289]. Onion extract contains quercetin and kaempferol, which inhibit fibroblast proliferation and collagen production by inhibiting TGF-β1, TGF-β2 and SMAD proteins [291]. Resveratrol inhibits collagen synthesis in KD fibroblasts, by downregulating HIF-1α [305]. Orally administered saireito can reduce postoperative edema after blepharoptosis surgery by suppressing TGF-β1-induced Smad 2/3 phosphorylation [254,314]. Shikonin inhibits the expression of p63, cytokeratin 10, α-SMA, TGF-β1, and collagen I [310]. Emodin exerts an anti-fibrotic effect by suppressing TGF-β1 signaling and subsequently inhibiting inflammation, myofibroblast differentiation and ECM deposition [316,318]. Glabridin can suppress the human KD fibroblast cells’ proliferation by inducing apoptosis and reducing collagen production [320]. Kaempferol inhibits TGF-β1/Smads signaling and inhibits fibroblast collagen synthesis, proliferation and activation in HTSs [322]. Tripterine inhibits proliferation and promotes the apoptosis of KD fibroblasts by inducing ROS generation and activating the JNK signalling pathway [323]. Wubeizi ointment suppressed KD formation by inhibiting fibroblast proliferation and promoting fibroblast apoptosis [326].
Statin Pravastatin Simvastatin	Pravastatin reduces the scar elevation index and decreases type I/III collagen content and myofibroblast persistence in the wound. Simvastatin is an effective inhibitor of the TGF-β1-induced production of type I collagen, connective tissue growth factor, and α-SMA production in KD fibroblasts [330,335,336].
Steroids Triamcinolone acetonide, Dexamethasone, Hydrocortisone acetate, Methylprednisolone	Triamcinolone acetonide is the criterion standard in the nonsurgical management of KDs and HTSs, and these glucocorticoids inhibit the cellular proliferation and production of collagen, glycosaminoglycan, hyaluronic acid, and TGF-β1 by dermal fibroblasts [340,344]. Other glucocorticoids such as dexamethasone, hydrocortisone and methylprednisolone also suppress VFGA expression.

ACE: angiotensin-converting enzyme. α-SMA: alpha-smooth muscle actin. ECM: extracellular matrix. EGCG: epigallocatechin gallate. HTSs: hypertrophic scars. INF: interferon. IL: interleukin. JNK: c-Jun N-terminal kinase. KD: keloids. MMP: matrix metalloproteinase. mRNA: *messenger RNA*. PBMC: peripheral blood mononuclear cell. RI: receptor I. RII: receptor II. ROS: reactive oxygen species. α-SMA: α-smooth muscle actin. TGF: transforming growth factor.TNF-α: tumor necrosis factor-α.

### 3.19. Combination Pharmacotherapy of Triamcinolone Acetonide with Other Drug(s)

It may be considered that the clinical efficacy of combination pharmacotherapy using multiple drugs could be superior compared to a monotherapy using each drug if the combination of drugs is pharmacologically adequate. An adequate combination pharmacotherapy can target multiple affected sites simultaneously, and exert additive and/or synergistic pharmacological action. For KD/HTS treatment, various combination pharmacotherapies are reported. In this section, some combination pharmacotherapies for KD/HTS treatment are reviewed.

#### 3.19.1. Combination of Triamcinolone Acetonide with 5-Fluorouracil

In modulating KD fibroblasts in vitro, TAC suppressed cell proliferation and induced G1 cell-cycle arrest, but not apoptosis. The addition of 5-FU to TAC improved scar regression and reduced the recurrence of KD [417]. This may suggest that the combined use of multiple drugs with different pharmacological actions can increase the treatment potency. In clinic, frequent injections of 5-FU are efficacious during the period of stabilization and resolution of the scars. The combination of TAC and 5-FU appeared to be more effective and less painful. The addition of the pulsed dye laser treatment simultaneously with injection therapy was found to be the most effective [418]. The clinical efficacy in treating KDs and HTSs was compared among three modalities: TAC alone (Group 1), the combination of TAC + 5-FU (Group 2), and TAC + 5-FU + pulsed-dye laser (PDL) (Group 3). The overall efficacy of TAC + 5-FU was comparable with that of TAC + 5-FU + PDL, but the combination of TAC + 5-FU + PDL was more acceptable to the patients, and produced better results [419]. Similarly, clinical efficacy in the treatment of KDs and HTSs was compared between the combination of intralesional TAC + 5-FU and the TAC monotherapy. The overall efficacy of the TAC +5-FU combination was comparable with that of TAC alone, but the combination was more acceptable to patients and produced better results [420]. Separately, another group compared the clinical efficacy of the combination of intralesional TAC + 5-FU with intralesional TAC alone in treating KD. The combination of TAC + 5-FU was superior to intralesional TAC monotherapy [421]. TAC suppresses cell proliferation and induces G1 cell-cycle arrest. 5-FU induces G2 cell-cycle arrest and apoptosis, and plays a predominant role in the combined treatment leading to more significant cell proliferation inhibition, apoptosis, type I collagen (Col-1) suppression and matrix MMP-2 induction [417]. In the treatment of KDs, scar improvement by ≥50% was found in most cases treated with 5-FU monotherapy, as well as with MMC, BLM and steroid injection monotherapy. Combined intralesional 5-FU + steroid injection produced statistically significant improvements. Monotherapy recurrence rates ranged from 0 to 47% for 5-FU, 0 to 15% for BLM and 0 to 50% for steroid injection. Combined therapy in the form of surgical excision and adjuvant 5-FU or steroid injections demonstrated lower recurrence rates; 19% and 6% respectively [99]. The clinical efficacy in the treatment of KD was compared among intralesional TAC + 5-FU, 5-FU + VER, enalapril alone, VER alone, and fractional carbon dioxide laser. The combination of TAC + 5-FU showed the greatest degree of scar softening and average size reduction, followed by 5-FU + VER; however, other modalities showed fewer effects, although all these treatments led to the resolution of pain and itching in the KD [422]. The efficacies of intralesional TAC + 5-FU (Treatment A) and intralesional TAC + BLM (Treatment B) in the treatment of small KD were compared. A greater improvement in the signs and symptoms of KD was obtained in Treatment B (excellent response 76%, a good response 10%, a fair response 6.66%, and a poor response 6.66% in 30 patients) compared to Treatment A (excellent response 50%, a good response 10%, a fair response 10%, and a poor response 6.66% in 30 patients). In addition, the VSS score and recurrence rates were statistically significant in Group B [423]. The clinical efficacy was compared between 5-FU alone and a combination of 5-FU + TAC in treating KD. 5-FU, both as a single agent and in combination with TAC, was equally efficacious in reducing the KD size, and the combination group reduced the side effects [424]. The efficacy and safety of a combination of TAC and 5-FU were compared with those of TAC alone and 5-FU alone in treating HTSs and KDs. The combination treatment of TAC and 5-FU showed higher efficacy than that of TAC alone and 5-FU alone [425]. In a review article, the efficacy and safety of TAC monotherapy and the combination therapy of TAC + 5-FU for treating HTS and KD were compared. The combination of TAC and 5-FU was more suitable for the treatment of HTSs and KSs, with greater improvement in scar height and patient satisfaction as well as fewer side effects [426,427]. Also, it was reported that a combination of intralesional TAC and 5-FU was more effective and safer than TAC monotherapy in the treatment of KD and HTS [90]. The combination of TAC + 5-FU was recommended for treating KDs not responding to silicone-based products, cryotherapy or intralesional corticosteroids alone. The objective data, such as those on height, volume, penetration depth of scars, POSAS and the Dermatology Life Quality Index (DLQI), of the combination treatment of 5-FU (50 mg/mL) and TAC suspension (40 mg/mL) (3:1) were evaluated for 12 months. The injected volume of drug suspension per KD was reduced with visiting time. The results of this study confirm the efficacy and safety of the combination of 5-FU and TAC in KD [428]. In a review article, it was reported that the combination of TAC with scar-modulating agents such as 5-FU, BLM, and BTX-A had an increased efficacy and fewer or similar adverse events. In particular, the combination of TAC and 5-FU showed the strongest and most consistent evidence out of all combinations supporting it use in HTS and KD treatments [429].

#### 3.19.2. Combination of Triamcinolone Acetonide with Verapamil

The efficacy of a combined regimen of calcium channel blocker (VER), steroid, and INF in treating HTSs was compared with steroid monotherapy in nude mice implanted with human HST fragments on their backs. The number of HTS fibroblasts in mice treated with a combined regimen significantly decreased at 10 days, as follows: (no drug) 16.6 × 10^5^; (steroid alone) 1.5 × 10^5^; and (combined regimen) 0.4 × 10^5^ (*p* < 0.05). Also, the fibroblast-populated collagen lattice (FPCL) contraction rates at 4 weeks were as follows: (no drug) 15.4%; (steroid alone) 65%; and (combined regimen) 73.4% of the original size, respectively (*p* < 0.05) [430]. The clinical efficacies of intralesional TAC alone, VER alone, and the combination of TAC and VER in treating KDs were compared. TAC showed a more significant and rapid improvement compared with VER, but this was associated with a higher rate of adverse effects. A combination of two drugs can augment their mechanisms without unwanted side effects [431]. The combination of TAC and VER incorporated in hydroxypropyl β-cyclodextrin (HP-β-CD) microneedles significantly decreases the thickness of HTSs, as well as hydroxyproline (HYP) and TGF-β1 expression in HTSs, and it improves collagen fiber arrangement and reduces dermis congestion and hyperplasia [432]. The clinical efficacy of intralesional triamcinolone alone and a combination of triamcinolone and VER was compared in KD patients. Both groups showed significant improvements, but the combination group saw the quicker resolution of skin redness and required fewer sessions [433].

#### 3.19.3. Combination of Triamcinolone Acetonide with Other Drugs (Bleomycin, Botulinum Toxin A, Interferon, Pentoxifylline, Platelet-Rich-Plasma)

A combination treatment using TAC (4 mg) and BLM (0.375 IU) applied to a 1 cm^2^ scar surface was an acceptable procedure in the treatment of KDs and HTSs [434]. The clinical efficacy in treating KDs and HTSs was compared between BLM tattoo and cryotherapy combined with intralesional TAC injection, in which the combination of cryotherapy and intralesional TAC injection was found to be the most common traditional therapy for HTSs and KDs. It was concluded that BLM tattoo may be more effective than cryotherapy combined with intralesional TAC injection in the treatment of larger KDs and HTSs (size > 100 mm^2^) [435]. Separately, the efficacy of the combined application of TAC and BLM for treating refractory KD and HTS was evaluated. The combined application effectively cured KDs and HTSs. Although large KDs showed local recurrence after treatment, the recurrence disappeared with further treatment [436]. The clinical efficacy of an intralesional TAC (13.3 mg/mL) and intralesional BLM (1 unit/mL) mixture used to treat refractory KD was examined. This combination offered a promising treatment option for individuals who have not responded well to traditional therapies, in which an excellent response was 78.8%, a fair response was 21.2%, and a poor response (<25% flattening) was 0%. Side effects such as ulceration, hyperpigmentation, hypopigmentation, secondary infection, and telangiectasis were observed [437]. In the treatment for ear KD with surgical shave excision followed by intralesional TAC and onabotulinum toxin A, 96–100% of patients expressed satisfaction (satisfied and very satisfied) [438]. Monotherapy with TAC (1 μg/mL) or IFN-γ (1.000–10.000 IU/mL) for 2 days induced a severe reduction in the proliferative potential in both normal healthy and KD fibroblasts in vitro. The combination of TAC and IFN-γ exhibited the stronger suppression of collagen type I synthesis in KD fibroblasts [439]. The efficacies of the combination of intralesional TAC and PRP (treatment A) and TAC alone (treatment B) were compared in the KD treatment. Treatment A yielded cosmetically better outcomes with a lower incidence of TAC-induced side effects, especially atrophy and hypopigmentation [440]. In the treatments of various scars with PRP, moderate improvement was the most frequently observed (36%) outcome when PRP was used alone. In contrast, when intralesional PRP was combined with laser or microneedling, most patients experienced marked (33% and 43%, respectively) or excellent (32% and 23%, respectively) results [441]. Significantly better improvements in height, pigmentation, pliability and overall VSS score were observed in patients who received intralesional PRP 1 week after TAC injections, as compared with TAC injections alone (intralesional TAC 20 mg/mL for four sessions, 3 weeks apart) [440]. In treating KD scars, the efficacy and safety of intralesional PTF alone, intralesional TAC alone and a combination of TAC and PTF were compared. A combination of TAC and PTF produced significantly better results for KD treatment, and lowered the risk of TAC-induced side effects, in which PTF monotherapy showed a lower efficacy than intralesional TAC [292]. In general, a combination pharmacotherapy shows greater clinical efficacy with fewer side effects. In review articles, the efficacy of combination pharmacotherapy was addressed. The efficacies of different injection and topical treatment strategies for HTSs and KDs were analyzed using various databases. The order of efficacy predicted by the surface under the cumulative ranking (SUCRA) curve was as follows: TAC + BTX-A (82.2%) > TAC + 5-FU (69.8%) > BTX-A (67.3%) > 5-FU + silicone gel (59.4%) > TAC + silicone gel (58.3%) > 5-FU (49.8%) > BLM (42.0%) > TAC (26.7%) > VER (26.2%) > silicone gel (18.3%). The authors recommended a combination therapy of the intralesional injection of TAC + BTX-A and TAC + 5-FU [442]. Separately, the combination of intralesional TAC+ BTX-A injection was reported to be the most effective for the treatment of KD and HTS, as follows: TAC + BTA > TAC + 5-FU > VER, BLM >BTX-A, TAC, 5-FU [443].

### 3.20. Combination of Pharmacotherapy with Physical Therapy

To treat KD/HTS scars, physical therapies such as cryotherapy, laser therapy, radiotherapy including brachytherapy, and silicone gel/sheeting are available. These physical therapies themselves are effective in treating KD and HTS. Furthermore, pharmacotherapy is sometimes combined with physical therapy, with or without surgical excision, to increase the clinical efficacy in treating KD/HTS [6,7,8,9,444].

#### 3.20.1. Pharmacotherapy Combined with Surgical Excision and Cryotherapy

The intralesional injection of liquid nitrogen in KD/HTS can freeze and necrotize the small regions of scars [445]. This therapy has only two minor disadvantages: histological controls cannot be performed, and the non-bloody artificial necrosis must be protected for 2–3 weeks by the local administration of antibiotics. Liquid nitrogen cryotherapy was reported to be superior to argon cryotherapy and CO_2_ laser therapy in the treatment of benign epidermal pigmented lesions [446]. Separately, cryotherapy with the use of an argon gas-based system proved to be effective in the treatment of KD, yielding volume reductions and low recurrence rates [447]. The argon gas device displayed a lower end temperature and a faster freezing rate in vivo compared to the liquid nitrogen device, but caused more hypopigmentation compared to the liquid nitrogen device following treatment [428]. Cryotherapy for the treatment of KD shows favorable results in terms of volume reduction and alleviated complaints of pain and pruritus. However, no complete scar eradication has been established, and recurrences are observed [448]. Treatment with the combined use of intralesional cryotherapy and postoperative silicone gel sheeting showed better results in terms of hardness, pain, and discomfort, as compared with cryotherapy alone [449]. The treatment of topical intralesional cryotherapy, applying liquid nitrogen intraoperatively to the inside of the skin flaps immediately after post-intralesional KD excision and before wound closure, showed a good aesthetic and symptomatic result [450]. Intralesional cryotherapy with liquid nitrogen was inferior to KD excision followed by brachytherapy for resistant KD. In primary KD, intralesional cryotherapy reduced KD volume. Thus, it could be used in KD patients and specific cases [445]. Combination KD therapy using superficial cryotherapy, fractional lasers, and intralesional TAC injection was safe and more effective than individual monotherapies [451]. Three cryotherapy sessions with two freezing–thawing cycles of 30–40 s freezing time and two minutes’ thawing time, undertaken one month apart, resulted in the complete flatness of the KD and no recurrence after 5 years [452].

Intralesional cryotherapy combined with intralesional TAC may yield the most promising results for non-auricular KD. It was reported that this should be considered a first-line treatment [453]. Other research groups reported that spray-type (−79 °C) cryotherapy was ineffective as a monotherapy, and should be used in combination with intralesional corticosteroids or BTX-A for favorable outcomes in the treatment of thick KD [454].

#### 3.20.2. Pharmacotherapy Combined with Surgical Excision and Laser Therapy

The efficacy of the treatment using a 585 nm flashlamp-pumped pulsed-dye laser (PDL) was comparable with those of intralesional 5-FU alone and a combination of corticosteroid and 5-FU [455]. The efficacies of TAC alone (Group 1), TAC + 5-FU (Group 2), and TAC + 5-FU and 585 nm flashlamp-pumped pulsed-dye laser (Group 3) were compared. The overall efficacy of Group 2 was comparable with that of Group 3, but the combination of Group 3 was more acceptable to the patients and produced better results [419]. Fractional ablative lasers create ablation zones at variable depths of the skin and enhance the transdermal delivery of substances. The combination of a fractional laser and immediate post-operative corticosteroid delivery in patients with HTSs resulted in average overall improvement [456]. A 2940 nm ablative fractional erbium laser was used, and betamethasone cream was topically applied twice a day under occlusion with transparent film dressings. The median percentage of improvement was 50%. The mean follow-up was 8 months, and the recurrence rate was 22% [457]. The combination therapy of a fractionated CO_2_ laser and laser-assisted drug delivery with topical TAC ointment for KD treatment was examined. This treatment showed excellent cosmetic results sustained at 22 months post-treatment [458]. Laser-assisted drug delivery (LADD) often uses ablative fractional lasers (CO_2_ or erbium: YAG lasers) because of their capacity to produce microscopic ablated channels. LADD is a promising technique that enhances the absorption of topical molecules such as corticosteroids, photosensitizers, and immunotherapy agents (imiquimod or 5-FU) while adding the synergistic effect of the laser [459]. The most widely used lasers are pulsed-dye lasers (PDLs) and fractional lasers. It was reported that, ideally, a combination approach using topical and intralesional medications along with a pulsed-dye laser and a fractional laser should be considered in all patients wishing to undergo treatment for their hypertrophic and atrophic scars. KD scars tend to be resistant to standard therapy, so other modalities should be considered [460]. Fractional CO_2_ lasers and intralesional verapamil are as efficient as intralesional triamcinolone for treating KDs, although it takes longer for the laser and verapamil to act compared to triamcinolone [461]. Laser-assisted 5-FU topical delivery tended to show a higher efficacy than laser-assist topical verapamil hydrochloride delivery, without significance, and TGF-β1 expression was significantly decreased after laser sessions [462,463]. BTX-A is a promising treatment for HTSs and KDs, and the combination with a fractional CO_2_ laser can enhance the efficacy of BTX-A in HTS treatment [464]. The fractional ablative lasers CO_2_ 10,600 nm and E r: YAG 2940 nm were found to produce the best results regarding erythema, height, and pliability, while the flashlamp-pumped pulsed dye laser (PDL) 585 nm scored slightly below that [465]. The combination of fractional CO_2_ and Nd-YAG lasers has a synergistic effect and is the most effective in the management of KD, with fractional CO_2_ being more effective than ND-YAG and Nd-YAG being the least effective [466]. In the case of PRP, the combination therapy of PRP with another drug or physical therapy appeared to be more effective compared with PRP monotherapy. PRP is an effective adjunct to ablative fractional CO_2_ lasers in treatments of burn-derived HTSs, and the combination of PRP and ablative fractional CO_2_ lasers proved to be more useful than ablative fractional CO_2_ lasers alone [467]. The clinical efficacy of a fractional CO_2_ laser versus a fractional CO_2_ laser accompanied with TAC or trichloroacetic acid was evaluated in KD patients. Significant and potent reductions in VSS score and KD scar thickness were observed as a result of the combination of the CO_2_ laser and TAC [448]. The therapeutic effect of the combination of a fractional CO_2_ laser and a topical TAC (Group A) was compared with that of intralesional TAC monotherapy (Group B) in treating KD. The KDs were completely resolved in 63.6% and 72.7% of the patients, and the rates of recurrence at 1 year were 9.1% and 18.2% in groups A and B, respectively [468]. The efficacies of a combination of cryotherapy with intralesional corticosteroid and a combination of fractional CO_2_ laser followed by topical corticosteroids for the treatment of KDs were compared. Both regimens showed excellent responses with minimum recurrence rates; however, the second regimen was aesthetically superior to the treatment [469]. Compared with TAC injection alone, the combination of pulsed dye laser and TAC shortened the relative cure time, reduced the number of TAC injections, and improved the clinical efficacy in the treatment of KD with post-operative recurrence [470].

#### 3.20.3. Pharmacotherapy Combined with Surgical Excision and Radiotherapy

Radiotherapy is one of the therapeutic methods available for KDs, and the irradiation technique has been developed from superficial X-ray to brachytherapy after decades of clinical practice [471,472]. External radiation following excision, often combined with other therapies, has been associated with recurrence rates of less than 10%. Various lasers have been used in the treatment of KD with great variability in their recurrence rate, but in general, the recurrence rates are similar to those of conventional surgery. As with cryo-destruction, laser ablation recurrence rates are often improved when combined with other treatments [473]. Following radiotherapy with >10 Gy, the recurrence rate of KDs decreased as a function of the biologically effective dose (BED) of the irradiation regimen, and for BED values > 30 Gy, the recurrence rate was <10%. The radiation treatment should be administered within 2 days of surgery [474]. The recurrence rates of KD after excision alone (*n* = 28), post-excision external beam radiotherapy (EBR), and interstitial high-dose-rate brachytherapy were 44%, 19% and 23%, respectively [475]. When brachytherapy with Iridium 192 was performed after a maximum of 7 h following surgery, the recurrence rate was 23.6%. One research group reported that only 1 recurrence out of 38 was found after 1 × 6 Gy + 2 × 4 Gy, and there were none after 3 × 6 Gy. Another group reported that treatment with a biologically equivalent dose >60 (20 Gy in five fractions) yielded superior local control over lower dose regimens. It was also reported that combination therapy—especially surgical excision with postoperative radiotherapy—was the best in preventing recurrence. After the excision of resistant KD, high-dose-rate brachytherapy with a biologically equivalent dose of approximately 20 Gy was recommended, in which the overall full recurrence rate was 8.3%. Combination therapy, especially surgical excision with postoperative radiotherapy, was reported to be the best in preventing recurrence [476]. The success rate of the treatment with complete excision, skin grafting, a one-time intraoperative injection of triamcinolone, immediate radiotherapy, and sustained pressure therapy was 87.5% at 1-year follow-up [477]. Intralesional TAC and 5-FU injections could effectively reduce the thickness and modify the hardness of small and young KDs. The combination of Strontium-90 brachytherapy with TAC and 5-FU could effectively reduce small-sized KD recurrence from 65.7% (without brachytherapy) to 44.4% after three intralesional TAC and 5-FU injections [478]. Brachytherapy and electron beam radiotherapy are the gold standards of treatment because brachytherapy provides a radiation treatment more focused on focal tissue in order to significantly reduce the recurrence rate and better preserve normal tissue [471]. Individualized surgery combined with early postoperative radiotherapy and TAC injection is an ideal treatment method that can ensure good auricular appearance, as well as low incidences of complications and recurrence, based on the effective treatment of auricular KD [479].

#### 3.20.4. Pharmacotherapy Combined with Surgical Excision and Silicone Gel/Sheeting

In KD/HTS treatment, the treatment efficacy of silicone was compared between a silicone gel-filled cushion and silicone gel sheeting. Both the cushion and the sheeting were effective [480]. It has been reported that silicone-based products, such as sheets and gels, are recommended as the gold standard, first-line, non-invasive option for both the prevention and treatment of scars. Silicone elastomer sheeting is an attractive treatment option because of its ease of use and low risk of adverse effects [481]. Silicone gel sheeting produced a statistically significant reduction in scar thickness and color amelioration in the treatment of KDs/HTSs [482]. When scar size, induration, and symptoms of KDs/HTSs were compared between silicone and non-silicone gel dressing groups after 4.5 months of application, there was no significant difference between the two treatment groups, and both dressings were shown to be equally effective in the treatment of KDs and HTSs [483]. The application of silicone gel sheeting significantly increased the mean baseline surface temperature of the HTS [484]. Silicone materials such as silicone occlusive sheeting and silicone gel sheeting can prevent HTS and KD formation. Regarding the action of silicone therapy, it is thought that the occlusion of the scar site causes the hydration of the wound bed and the suppression of the overactivity of scar-related cells [485,486]. In addition, this physical therapy can improve scar color, size, erythema, pliability, pain, and itching, due to the hydration of the skin’s corneal layer and the modulation of the cell signaling between fibroblasts and keratinocytes, mediated by cytokines [482]. In particular, the early application of a silicone sheet yields a significant improvement in the prevention of postoperative scarring [487]. The clinical efficacy of using topical silicone gel and topical methylprednisolone cream in preventing HTS and KD formation following Pfannenstiel incisions was evaluated. Scores regarding height, pigmentation, vascularity, pliability, and total modified VSS significantly decreased in all groups (control, silicone, and methylprednisolone groups). Patient satisfaction was higher in the methylprednisolone group [488].

### 3.21. Prevention of Recurrence of KD/HTS Scars after Surgical Excision

The growth of KDs continues with time, and spontaneous regression is not expected, which is different from the case of HTSs [25] (Table 1). To treat KD scars completely, surgical excision may be essential. However, the recurrence rate of KDs after the surgical excision of KDs without preventive treatment is quite high, at 50–100% [26,27,474]. When the recurrence rates of KDs were compared between excision alone (control group) and postoperative injection with TAC or IFN-α2b, the recurrence rates were 51.1% in the control group, 58.4% in the TAC group and 18.7% in the IFN-α2b group, indicating that TAC could not suppress the recurrence rate [411]. In contrast, other researchers reported that the recurrence rate of KD after the intralesional injection of IFN-α2b was 54%, and that after intralesional triamcinolone was 15%. The authors concluded that IFN-α2b does not appear to be effective in the clinical management of KD [489]. The topical application of MMC was reported to decrease the postoperative recurrence rate of KD in the head and neck to 10% (1 out of 10 patients) [444]. Topical imiquimod has also been used in preventing the recurrence of KD after surgical excision. Shaved KDs were treated with imiquimod 5% cream or vehicle cream nightly for two weeks, and three times a week under occlusion for one month. At 6 months, the KD recurrence rates were 37.5% (3/8) in the imiquimod group and 75% (3/4) in the vehicle group (*p* = 0.54) [490]. The injection of a porcine gelatin dextran hydrogel scaffold was examined for its effects on the prevention or reduction of recurrence of KD scars after the surgical excision of ear KDs. The recurrence rate after surgical excision alone was 51.2% at 1 year. In contrast, the recurrence rate associated with the hydrogel scaffold was 19.2%, which is statistically superior to the historical recurrence rates (50–100%) given in the literature [26]. One KD was excised per subject, and each wound was half randomized to receive intralesional injections of TAC (10 mg/mL) or VER (2.5 mg/mL) at monthly intervals (four doses). Data analysis demonstrated significantly higher KD recurrence with VER compared to TAC at 12 months post-surgery [409]. The recurrence rate after the surgical excision of KD was 16.5% in the topical MMC group, and 24.7% in the imiquimod 5% cream group, indicating that both topical MMC and imiquimod are effective alternatives in preventing KD recurrence [491]. The effects of surgical excision and TAC injection immediately after the surgical removal of old caesarean section KD scars on the recurrence of the scars were evaluated in pregnant women with KD scars. Patients received surgical excision and TAC injection sub-dermally at the time of wound closure after the delivery of the baby. It was concluded that this combination therapy will be safe, long-lasting and cost-effective when used in the management of caesarean KD scars [348]. The rate of recurrence after surgical excision with perioperative TAC injections was evaluated in patients with recurrent auricular KDs. The recurrence rate was 9.6% during the 13-month (average) follow-up period [492]. The 5-year recurrence rate of combination (intraoperative/postoperative TAC + intraoperative PRP injections combined with surgical excision) was significantly lower when compared with other groups (intralesional TAC injections alone, intraoperative/postoperative TAC injections combined with surgical excision). It was concluded that this combination (surgical excision +TAC + PRP) in patients with ear KDs is a highly successful multimodal treatment in terms of low recurrence and adverse effects [493]. Regarding the recurrence rates of scars in the treatment of KDs and HTSs with intralesional TAC and 5-FU, the recurrence rates reported were 7.5–23.3% when the follow-up period was approximately 11 months (range 1–24 months). In contrast, however, six studies using various intralesional combination regimens reported 0% recurrence over the follow-up period (TAC + 5FU, TAC + BTX-A, TAC + BLM, TAC+ cryotherapy). A consistent approach to recurrence rate reporting across studies was lacking, and long-term follow-up (18–24 months) is needed to characterize recurrence in the treatment of pathologic scars using various intralesional agents [494]. To prevent the recurrence of KDs, surgical excision, compressive therapy, silicon dressings, corticosteroid injections, radiation, cryotherapy, INF therapy, and laser therapy have all been used alone or in combination. Despite this wide range of available treatments, recurrence rates typically remain in the 50–70% range [444]. The effectiveness of silicone gel and topical tretinoin cream for the prevention of HTSs and KDs following surgical excision was evaluated. Both treatments effectively prevented the recurrence of HTS/KD and improved scars after surgery [495]. The efficacy of a silicone gel containing onion extract and aloe vera compared to silicone gel sheets was assessed regarding its ability to prevent postoperative HTSs and KDs. After the 12-week follow-up, there were no statistically significant differences in the scarring incidence rates of both groups, indicating that both treatments are as effective as silicone gel sheets in postoperative scar prevention [262]. The efficacy of surgical excision combined with intralesional PRP and postoperative in-office superficial radiation therapy in preventing KD recurrence was evaluated in patients with KD scars. This combination therapy achieved a 95.5% nonrecurrence rate at 1- to 3-month follow-up [496]. The total recurrence rates were 29.3% when treated with surgical removal followed by irradiation at 15 Gy and 14.0% after 2003 when treated with electron beam irradiation at total doses of 10, 15, or 20 Gy, depending on the site. It was suggested that KDs and intractable HTSs with a high risk of recurrence should be treated with 20 Gy in four fractions over 4 days, and that earlobes should be treated with 10 Gy in two fractions over 2 days [497]. In patients who underwent a median sternotomy, a silicone gel sheet was kept directly on the surgical incision for 24 h starting 2 weeks after surgery, and then repeated every 4 weeks for 24 weeks with a new sheet. None of the patients experienced an aggravation of any subjective symptoms during the 24-week study, or even after 24 weeks [498]. Patients with auricular KD were treated by triple combination therapy: surgical excision, PRP, and cryosurgery. This combination therapy was effective in treating auricular KD, with a low recurrence rate and a favorable cosmetic outcome [499].

## 4. Discussion

In this article, at first, we tried to evaluate the clinical efficacy or the rank order of usefulness of mono-pharmacotherapy using a single drug in treating KD/HTS. However, putting each monotherapy in order is not easy, or almost impossible, because the reported clinical efficacies of each monotherapy are not consistent, but rather differ according to different researchers. Different from the case of in vitro studies, the in vivo clinical conditions are greatly variable among patients. In general, the pharmacological actions of drugs are dose-dependent at least in in vitro conditions, indicating that they can exhibit appropriate pharmacological action only at an appropriate concentration. At a lower concentration, each drug exerts no effective pharmacological action and induces unwanted side effects at a higher concentration. In in vivo clinical situations, it will be very difficult to settle on the appropriate concentration of each drug to be applied in KD/HTS scar tissues. In addition, many other factors, such as variations in the symptoms among patients, the starting time of pharmacotherapy, dose and dosing formulations of the drug, treating frequency, treatment period, treating techniques such as intralesional injection and/or topical application, the timing of evaluation of drug efficacy, and the method of evaluation (symptoms) of efficacy (for example, size of scars, pain/itching, satisfaction of patients), also greatly affect the evaluation of clinical efficacy. In this section, some factors are discussed.

### 4.1. Effect of the Starting Time of Pharmacotherapy

Recurrence rates of excised KDs were compared between treatments involving the postoperative injection of TAC and those using INF-α2b. The postoperative injection of INF-α2b to KD excision sites offered a therapeutic advantage over KD excision, although postoperative TAC injections did not reduce the number of KD recurrences [411]. The effects of topical silicone gel and methylprednisolone cream in preventing HTS and KD formation following Pfannenstiel incisions were compared in the 3rd and 6th months. The use of topical methylprednisolone cream in fresh wounds in the early postoperative period (3rd month) appears to be promising. No side effects were experienced by the patients with either treatment, and patient satisfaction was higher in the methylprednisolone group [488]. The efficacy of intralesional TAC injection was compared between the early stage (≤6 months after injury) and the static stage (>6 months after injury). TAC treatment improved the color, thickness, softness, vascular distribution and hardness of HTS/KD scars. The efficacy of TAC treatment was better when it was applied during the static stage of pathological scarring, rather than in the early stage [500]. In a systemic review, it was reported that TAC may be beneficial for use in the short-term treatment of HTS and KD; however, 5-FU, TAC + 5-FU, and VER may produce superior results in medium- and long-term treatments [501]. The starting time of pharmacotherapy and the time of evaluation are very important when evaluating the clinical efficacy of each type of pharmacotherapy, including combined pharmacotherapy.

### 4.2. Effect of the Dose and Dosing Formulation

The clinical efficacy of intralesional TAC injection on KD treatment was evaluated. Intralesional TAC injection yielded a good result, and the TAC dose of 7.5 mg/cm^2^ showed better efficacy than the dose of 15 mg/cm^2^ [104]. In a rabbit ear HTS model, the treatment was performed with intralesional simvastatin, lovastatin, or pravastatin at low, medium, or high doses on post-wounding days 15, 20, and 25. Low-dose (40 μM) simvastatin, lovastatin, and pravastatin each significantly reduced scar elevation by 21.9%, 25.8%, and 22.8%, respectively, and low-dose simvastatin also facilitated a significant reduction in CTGF expression. In contrast, medium- (120 μM) and high-dose (400 μM) statin groups did not change the scar elevation significantly [331]. Using three different TAC ointments (TA-A, a brand-name preparation, and TA-B and TA-C, two generics), some physicochemical properties such as cohesiveness, spreadability, production of TAC crystallization, oil/water contents, and viscoelasticity were compared. It was reported that differences in the types and contents of additives caused differences in the physicochemical properties of individual ointments [502]. It could be speculated that differences in the physicochemical properties of the three different TAC ointments also cause differences in clinical efficacies among them. The percutaneous permeability of TAC-lipid nanoparticles (TAC-LNPs with 232.2 ± 8.2 nm in size) in scar tissue was examined in vitro using the HTS scar tissue from a rabbit ear. The penetration into scar tissue of TAA-LNPs was 2-fold and 40-fold higher than that of common liposome and commercial suspensions, respectively [503]. The concentrations of each drug in scar tissues could vary among different clinical situations, although each drug has an optimal concentration at which it exerts the appropriate pharmacological action.

### 4.3. Effect of Evaluation Method of Clinical Efficacy

In treating KD scars, the clinical efficacy of intralesional TAC (4 mg/cm^2^ or 0.1 mL/cm^2^ of TAC 40 mg/mL, 3-week interval) was compared with that of 5-FU (10 mg/cm^2^ or 0.2 mL/cm^2^ of 5-FU 50 mg/mL, 3-week interval). 5-FU was more effective in resolving KD scars. The patient satisfaction score was higher in the TAC group, but the VSS score and VAS score were better in the 5-FU group [504]. When the clinical efficacies of using intralesional BTX-A and 5-FU in KD treatment were compared, BTX-A achieved the excellent flattening of the lesions, much better than 5-FU. In the BTX-A group, there was no statistically significant difference between the clinical response in small lesions compared to those in medium and large ones, although, in the 5-FU treatment, small and medium lesions showed significantly better responses than larger ones [219]. The clinical efficacies of intralesional TAC alone, VER alone, and the combination of TAC and VER in treating KD were compared. TAC yields a more significant and rapid improvement compared with VER, but is associated with a higher rate of adverse effects. A combination of two drugs can augment the mechanisms of each without unwanted side effects [431]. The main pharmacological actions of drugs are different among different drugs.

### 4.4. Effect of the Timing of Evaluation of Clinical Efficacy

The clinical efficacy in managing HTSs and KDs was compared between intralesional TAC and VER. Both drugs reduced the vascularity, pliability, height and width of the scar after 3 weeks and one year of follow-up, but not the pigmentation and length of the scar. Adverse drug reactions were more common with TAC than with VER [93]. The clinical efficacy of intralesional BLM for the treatment of KDs was evaluated. Complete flattening occurred at a rate of 70.8% and highly significant flattening occurred in 8.3%, in which local side effects included pains (100%), blisters (78.3%), ulceration (5.8%), and hyperpigmentation (56.7%). The recurrence rates of KDs 6, 12, 15, and 18 months after the last treatment were 3.8, 15.4, 45.5, and 50%, respectively [104]. In a review article, it was reported that in treating KDs and HTSs, TAC was more effective than VER in improving vascularity within 9 weeks. Also, TAC produced superior results related to improving pliability within 18 weeks, whereas VER produced superior results at between 18 and 24 weeks of treatment [92]. It was reported that a consistent mode of reporting recurrence rates across studies was lacking, and long-term follow-up (18–24 months) is needed to characterize recurrence in the treatment of pathologic scars using various intralesional agents [494]. As reported, the evaluations of clinical efficacy greatly differ depending on the timing of the evaluation. Ideally, the complete healing of KD/HTS scars is desired.

### 4.5. Effect of Treatment Techniques

The intralesional multi-injection of a therapeutic drug using a syringe is one of the most widely used and effective treatments for KDs and HTSs, because it enables the delivery of a therapeutic drug to the target site at a designated dose/concentration. However, the efficacy of intralesional injection using a needle is highly dependent on the skill of the medical professionals administering the injection [505]. Intralesional injection causes pain to the patients. Various administration methods of therapeutic drugs, including the enhancement of transdermal delivery, have been developed to enable targeted delivery with reduced pain, as follows: the co-injection of lidocaine using an electric syringe [506], the topical application of lidocaine [507,508], the use of cryoanesthesia [509], the use of a needle-free pneumatic jet-injection [120], microneedle patches [510] and so on. The transdermal iontophoretic delivery of medicine will not cause any pain, and the dosage formulations for poorly soluble compounds like corticosteroids can be modified as follows: use of a mixture of organic solvent and water [511], using nanostructured lipid carriers as vehicles [512], and using solid lipid nanoparticles hydrogel [513]. As well, the transdermal delivery of corticosteroids in a gel formulation was also reported for phonophoresis and ultrasound delivery [514]. Separately, the influence of surgical margins on the recurrence of HTSs has been investigated by dividing patients into two groups: the intramarginal excision group and the extramarginal excision group. All patients (15/15) treated with intramarginal excision experienced HTS recurrence within six months. Three of nine patients (33.3%) exhibited recurrence when the HTSs were excised with a 3–5 mm margin [514]. An efficient margin would be necessary to prevent the recurrence of HTSs.

## 5. Conclusions

We reviewed the use of pharmacotherapy for treating and/or preventing the postoperative recurrence of KDs/HTSs. Both KDs and HTSs are raised and pigmented scars with increased vascularization and cellularity, and these scars are formed due to the overproduction of fibrosis-enhancing cytokines such as TGF-β1/β2, and ECM such as collagen. These scars cause pain, itching, contracture, discomfort and so on, and greatly decrease the QOL of patients. Thus, clinically useful drugs can suppress the proliferation of fibroblasts, the production and/or function of TGF-β and ECMs (mainly collagen), vascularity, and/or pain and itching (Table 2). To increase the clinical efficacy of pharmacotherapy in treating KDs/HTSs, the appropriate selection of drug(s) depending on the patient’s symptoms (such as the size and site of scars), dosing factors (such as dose, dosing frequency and period, formulations, dosing timing for wounds, and dosage technique for topical and/or intralesional injection), and the method of evaluation of symptoms, including timings, would be very important. In general, combination pharmacotherapy is much more effective in treating and/or preventing the recurrence of KDs/HTSs, compared to mono-pharmacotherapy. The greater efficacy of combination therapy is due to its multiple targeting of affected sites simultaneously. By using a combination pharmacotherapy, including an appropriate physical therapy, additive and/or synergic pharmacological effects with fewer side effects can be expected. At present, KDs and HTSs are still intractable diseases. However, information on various drugs, their usage methods in patients, and clinical outcomes, together with evaluation methods, would be valuable when developing safer and more effective pharmacotherapies for KDs and HTSs.

## Figures and Tables

**Table 1:** Characteristics of keloid and hypertrophic scars.

	Keloids (KDs)	Hypertrophic Scars (HTSs)	Refs.
Common future	During the healing process of the cutaneous injury, growth factors such as TGF-β are released and they activate fibroblasts. The overexpression of TGF-β1/β2 and ECM such as collagen can cause raised and firm scars with increased vascularization and cellularity.		[3,12]
Onset	KD typically arise from 3 to 12 months, or from 3 months to several years.	HTSs appear within 1 month, or 4–8 weeks, of injury and grow over 6–8 months.	[13]
Scar formation	The raised scar of the KD extends beyond the boundaries of the original wounds.	The raised scar of the HTS is contained within the boundaries of the original injury.	[13,14]
Scar sites	Predominant anatomical sites of KDs are chest, shoulder, upper back, posterior neck, cheeks, knees, and earlobes.	No predominant anatomical sites of HTSs.	[16,17]
Incidence	Less common. Association with age ≥ 22 years, family history and genetic factors.	More common in males than females.	[15]
Genetic factor	A genetic predisposition is associated with skin color and familial disposition: African > Asian and Hispanic >> Caucasian populations.	No evidence of genetic factors’ influence. Less association between skin color and familial disposition.	[17,18,19]
Collagen	Approximately 20-fold higher collagen production. Increase in type I/III collagen ratio in parallel with the increase in α1(I) procollagen mRNA compared to normal skin tissue.	Approximately 3-fold higher collagen production than normal scars. A higher collagen III scar/normal ratio, with no difference in collagen I scar/normal ratio.	[11,12,22,23]
Proinflammatory factors	Upregulation of interleukin (IL)-1α, IL-1β, IL-6, and tumor necrosis factor (TNF)-α expression levels.	Increase in IL-31, IL-31 receptor α, and oncostatin M receptor expression levels.	[3,24]
Regression	KD growth continues with time and there is no spontaneous regression.	HTS growth is limited for months, and regression (contraction) occurs spontaneously.	[25]
Recurrence after surgical excision	The recurrence rate of KDs is high, 50–100%, after surgical excision without preventive treatments. If excised, preventive treatment is necessary.	The recurrence rate of HTSs is none or low after surgical excision.	[26,27]
